# Current Evidence of Measurement Properties of Physical Activity Questionnaires for Older Adults: An Updated Systematic Review

**DOI:** 10.1007/s40279-020-01268-x

**Published:** 2020-03-03

**Authors:** Matteo C. Sattler, Johannes Jaunig, Christoph Tösch, Estelle D. Watson, Lidwine B. Mokkink, Pavel Dietz, Mireille N. M. van Poppel

**Affiliations:** 1 Institute of Sport Science, University of Graz, Graz, Austria; 2 School of Therapeutic Sciences, Faculty of Health Sciences, University of Witwatersrand, Johannesburg, South Africa; 3 Department of Epidemiology and Biostatistics, Amsterdam University Medical Centers, Vrije Universiteit Amsterdam, Amsterdam Public Health Research Institute, Amsterdam, The Netherlands; 4 Institute of Occupational, Social and Environmental Medicine, University Medical Centre, University of Mainz, Mainz, Germany; 5 Department of Public and Occupational Health, Amsterdam University Medical Centers, Vrije Universiteit Amsterdam, Amsterdam Public Health Research Institute, Amsterdam, The Netherlands

## Abstract

**Background:**

Questionnaires provide valuable information about physical activity (PA) behaviors in older adults. Until now, no firm recommendations for the most qualified questionnaires for older adults have been provided.

**Objectives:**

This review is an update of a previous systematic review, published in 2010, and aims to summarize, appraise and compare the measurement properties of all available self-administered questionnaires assessing PA in older adults.

**Methods:**

We included the articles evaluated in the previous review and conducted a new search in PubMed, Embase, and SPORTDiscus from September 2008 to December 2019, using the following inclusion criteria (1) the purpose of the study was to evaluate at least one measurement property (reliability, measurement error, hypothesis testing for construct validity, responsiveness) of a self-administered questionnaire; (2) the questionnaire intended to measure PA; (3) the questionnaire covered at least one domain of PA; (4) the study was performed in the general, healthy population of older adults; (5) the mean age of the study population was > 55 years; and (6) the article was published in English. Based on the Quality Assessment of Physical Activity Questionnaires (QAPAQ) checklist, we evaluated the quality and results of the studies. The content validity of all included questionnaires was also evaluated using the reviewers’ rating. The quality of the body of evidence was evaluated for the overall construct of each questionnaire (e.g., total PA), moderate-to-vigorous physical activity (MVPA) and walking using a modified Grading of Recommendation, Assessment, Development, and Evaluation (GRADE) approach.

**Results:**

In total, 56 articles on 40 different questionnaires (14 from the previous review and 26 from the update) were included. Reliability was assessed for 22, measurement error for four and hypotheses testing for construct validity for 38 different questionnaires. Evidence for responsiveness was available for one questionnaire. For many questionnaires, only one measurement property was assessed in only a single study. Sufficient content validity was considered for 22 questionnaires. All questionnaires displayed large measurement errors. Only versions of two questionnaires showed both sufficient reliability and hypotheses testing for construct validity, namely the Physical Activity Scale for the Elderly (PASE; English version, Turkish version) for the assessment of total PA, and the Physical Activity and Sedentary Behavior Questionnaire (PASB-Q; English version) for the assessment of MVPA. The quality of evidence for these results ranged from very low to high.

**Conclusions:**

Until more high-quality evidence is available, we recommend the PASE for measuring total PA and the PASB-Q for measuring MVPA in older adults. However, they are not equally qualified among different languages. Future studies on the most promising questionnaires should cover all relevant measurement properties. We recommend using and improving existing PA questionnaires—instead of developing new ones—and considering the strengths and weaknesses of each PA measurement instrument for a particular purpose.

**Electronic supplementary material:**

The online version of this article (10.1007/s40279-020-01268-x) contains supplementary material, which is available to authorized users.

## Key Points

**Table:** 

Based on low-to-moderate-quality evidence of both sufficient reliability and hypotheses testing for construct validity, we recommend using the Physical Activity Scale for the Elderly (PASE—English version) for the assessment of total PA and the Physical Activity and Sedentary Behavior Questionnaire (PASB-Q—English version) for the assessment of MVPA.
To ensure high quality of and comparability across studies, we recommend using and improving existing questionnaires, rather than developing new versions, as well as evaluating strengths and weaknesses of each PA measurement instrument with respect to the study purpose.
We recommend performing high-quality studies on the most promising questionnaires, including an assessment of content validity and responsiveness, and the use of standards for study design and evaluation (e.g., COnsensus-based Standards for the selection of health Measurement INstruments (COSMIN) checklists).

## Introduction

The aging of the world’s population represents one of the key challenges over the next decades. Both life expectancy and the proportion of older adults are increasing [1] and, therefore, promoting and maintaining quality of life at an older age is essential. Current evidence shows that physical activity (PA) can increase health in later life [2] through increasing quality of life [3, 4], cognitive and physical functioning [5, 6] and decreasing the risks for neurodegenerative diseases (e.g., Alzheimer’s disease, vascular dementia) [7], depressive symptoms [8, 9] and all-cause mortality [10].

Several instruments are available to measure PA in older adults such as questionnaires, diaries, accelerometers and pedometers. Although several aspects (e.g., strengths, weaknesses and practical considerations) have to be considered when selecting an instrument [11], questionnaires appear to be popular for the measurement of PA in older adults [12]. In contrast to accelerometers, they are usually feasible in large epidemiological studies and well accepted by participants. For example, questionnaires are used in large national surveys to determine and compare PA levels among different countries [13]. The use of the same measurement method in these surveys facilitates comparability among PA estimates [14]. Furthermore, in addition to the total volume of PA, questionnaires can provide valuable information about different domains (e.g., home, leisure time) and types (e.g., walking, resistance training) of activities [15]. Finally, questionnaires can be used as a screening tool to determine PA levels of individuals in healthcare settings. The assessment can be integrated into the clinical workflow and linked to electronic record systems, whereas the obtained results can be used for counseling and PA promotion [16, 17].

Both researchers and healthcare professionals should use instruments with high measurement quality. The quality of an instrument is determined by evaluating its’ measurement properties such as reliability, validity and responsiveness. Sufficient measurement properties are indispensable to trust the results of studies on the efficacy of PA interventions, health benefits of PA, dose–response relationships as well as trends of PA over time. However, many PA questionnaires and modified versions of these have been developed. The great number of available questionnaires makes it difficult to choose the instrument with the best measurement properties. Moreover, the use of different questionnaires decreases the comparability of PA estimates and its relationship with health outcomes across studies and countries. To limit methodological biases and to draw study conclusions with the highest quality, it is important to select the questionnaire with the best measurement properties for a particular purpose.

Already in 2000, Sallis and Saelens [15] recognized a profusion of PA questionnaires and suggested to select only a few, most qualified ones for future studies. Existing reviews on measurement properties of PA self-reports [18–28] usually focused on the adult population or a specific population of older adults (e.g., older adults with dementia). However, although research on PA in older adults has grown continuously [2], no firm recommendations for the most-qualified self-administered PA questionnaires for older adults have been provided.

In 2010, a series of systematic reviews on measurement properties of PA questionnaires in youth [29], adults [30] and older adults [28] were published. Regarding older adults, we concluded that the evidence for measurement properties of PA questionnaires is scarce and future high-quality validation studies are needed. Specifically, the reliability of the Physical Activity Scale for the Elderly (PASE) was rated as sufficient but the results for validity were inconsistent. Recently, the review for youth was updated [19] and a new one for pregnancy was published [18]. The present review is an update for older adults and aims to summarize, compare and appraise the measurement properties (i.e., reliability, measurement error, hypotheses testing for construct validity, responsiveness) of all available self-administered PA questionnaires in older adults aged > 55 years. In addition, we evaluated the content validity of all included questionnaires and aimed to provide recommendations for choosing the best available PA questionnaires in older adults.

## Methods

For reporting, we followed the Preferred Items for Systematic Reviews and Meta-Analyses (PRISMA) guidelines [31]. A definition of all quoted measurement properties is provided in Table 1.

**Table 1:** Definition of measurement properties for PA questionnaires, adapted from the COSMIN methodology [135] (p. 743)

Domain	Measurement property	Aspect	Definition
Reliability			The degree to which the measurement is free from measurement error
	Internal consistency		The degree of the interrelatedness among the items
	Reliability		The proportion of the total variance in the measurements which is because of true differences among participants
	Measurement error		The systematic and random error of a participant’s score that is not attributed to true changes in the construct
Validity			The degree to which an instrument measures the construct it purports to measure
	Content validity		The degree to which the content of an instrument is an adequate reflection of the construct
		Face validity	The degree to which the items of an instrument indeed look as though they are an adequate reflection of the construct
	Construct validity		The degree to which the scores of an instrument are consistent with hypotheses (for example with respect to internal relationships, relationships to scores of other instruments) based on the assumption that the instrument validly measures the construct
		Structural validity	The degree to which the scores of an instrument are an adequate reflection of the dimensionality of the construct
		Hypotheses testing	Idem construct validity
		Cross-cultural validity	The degree to which the performance of the items on a translated or culturally adapted instrument are an adequate reflection of the performance of the items of the original version of the instrument
	Criterion validity		The degree to which the scores of an instrument are an adequate reflection of a gold standard
Responsiveness			The ability of an instrument to detect change over time in the construct
	Responsiveness		Idem responsiveness

*COSMIN* COnsensus-based Standards for the selection of health Measurement INstruments, *PA* physical activity

### Literature Search

We performed systematic literature searches in the databases PubMed, SPORTDiscus and Embase (using the filter ‘Embase only’). The search strategy involved (variations of) the terms ‘physical activity’, ‘questionnaire’ and ‘measurement properties’ [32] (see Electronic Supplementary Material Appendix S1). We excluded publication types such as case reports, interviews or biographies and adapted our search for Embase and SPORTDiscus following their guidelines. In 2010 [28], we included all publications until May 2009 in the initial title/abstract search. For this update, to avoid any losses of publications, we considered all results from September 2008 to 17 December 2018 (day of search) as potentially relevant. The search was updated on 3 December 2019.

### Eligibility Criteria

The following eligibility criteria were defined [18, 28, 33]:

1. The purpose of the study was to evaluate at least one of the following measurement properties of a self-administered questionnaire: reliability, measurement error, hypotheses testing for construct validity or responsiveness. Because no gold standard exists to measure PA [25, 34], results from studies referring to the criterion validity of a questionnaire were considered as evidence for hypotheses testing for construct validity.
2. The purpose of the questionnaire was to assess PA, which was defined as any bodily movement produced by skeletal muscles which results in energy expenditure (EE; p. 126) [35].
3. The questionnaire should cover at least one domain of PA (household, occupation, recreation, sports or transport [cycling and/or walking]).
4. The study was performed in the general population of older adults (i.e., healthy older adults), regardless of the population for which the questionnaire was developed (e.g., general population, patients with cardiovascular disease).
5. The mean or median age of the study population was > 55 years.
6. The article was published in English.

Consistent with our previous review [18], we did not evaluate measurement properties regarding the internal structure of the questionnaire (structural validity, internal consistency (e.g., using Cronbach’s alpha), cross-cultural validity). Internal structure is only relevant for questionnaires based on a reflective model assuming items to be correlated [33]. This is not the case for PA questionnaires (e.g., time spent in walking does not necessarily have to correlate with time spent in other behaviors) [36]. In addition, we did not perform an exhaustive evaluation of content validity but rather applied a subjective rating to assess the content validity of all included questionnaires [33]. A detailed evaluation of content validity may be performed in future reviews and would require the inclusion of all studies focusing on any aspect of content validity (e.g., studies on the development of the questionnaire, pilot tests among older adults, expert opinions).

Finally, the following exclusion criteria were applied:

1. Questionnaires measuring physical functioning or sweating, diaries, interviews (face-to-face, telephone), and interviewer-administered questionnaires. However, we did include self-administered PA questionnaires where some participants had received help with the completion.
2. Questionnaires assessing specific behaviors within one domain of PA (e.g., commuting to work).
3. Studies performed solely in patients or in a priori defined subpopulations (e.g., stroke patients, obese older adults).
4. Studies assessing the agreement between a PA questionnaire and a non-PA measure such as body mass index (BMI), health functioning, performance, fitness, wellbeing or cardiovascular risk factors. This was done because we found it difficult to define specific cut points for sufficient measurement properties.

### Selection of Articles and Data Extraction

Two researchers independently screened titles and abstracts for eligible studies. MCS and either CT or JJ inspected full-text articles, performed data extraction, result rating and quality assessment. Disagreements were discussed during consensus meetings. If no agreement could be reached, a third researcher (LBM, MVP) was consulted. Consistent with our previous reviews [18, 28], we extracted all relevant information using a standardized form. This form was based on the Quality Assessment of Physical Activity Questionnaire (QAPAQ) checklist [36]. We included the results for the overall construct of PA [i.e., total PA, total physical activity energy expenditure (PAEE)] and for any subdimension (e.g., leisure time physical activity (LTPA), moderate-to-vigorous physical activity (MVPA), walking) in our tables for which information about at least one measurement property was available. It is important note that, depending on the purpose of the questionnaire (overall construct), the total score of the questionnaire can either represent total PA, total PAEE or a specific subdimension of PA. For example, a questionnaire may aim in assessing LTPA and, hence, the total score of the questionnaire does not necessarily represent total PA.

### Assessment of Measurement Properties

Each result on a measurement property was either rated as sufficient (+) or insufficient (−). Our criteria for sufficient measurement properties were based on the QAPAQ checklist [36] and have been described previously [18, 28, 30]. However, a short description will be provided herein. The content validity of all included questionnaires was assessed following the reviewers’ ratings on three principal criteria [18, 30]: (1) If the questionnaire measures total PA (or MVPA), it should at least include the domains of household, recreation, sports and transport. Regarding transport, at least walking should be included since it represents one of the most common activities in older adults [37]. Occupational PA was considered as optional for older adults; (2) the questionnaire should assess at least the parameters frequency and duration of PA (e.g., to further define dose–response patterns between PA and health [38]); and (3) the recall period should be at least one week (if not assessing daily PA).

We included results for reliability [intraclass correlation coefficient (ICC), concordance, kappa, Pearson/Spearman correlation] and measurement error [coefficient of variation (CV), standard error of measurement (SEM), smallest detectable change (SDC), change in the mean or mean difference ($\bar{d}$; systematic error), limits of agreement (LOA; random error)]. Previous research has shown that already low doses of PA (e.g., < 150 min of MVPA, 1–2 times running per week) were associated with substantial health benefits in older adults such as reductions in all-cause mortality [10, 39]. Therefore, we defined a change in the frequency of two times per week and a change in MVPA of 30 min [≥ 90 metabolic equivalent (MET) minutes] per week as clinically important [18]. These values represent a minimal important change (MIC) and were used to evaluate measurement error. If the LOA or SDC are smaller than the MIC, changes as large as the MIC represent true changes beyond measurement error. In other words, a PA questionnaire should be able to measure changes of ± 20% of current PA guidelines [2].

A result for reliability was sufficient if ICC/kappa/concordance was ≥ 0.70 or Pearson/Spearman ≥ 0.80 and a result for measurement error if MIC (e.g., 30 min of MVPA per week) > LOA/SDC or CV ≤ 15%. Otherwise, the result was insufficient. Cut points for sufficient hypotheses testing for construct validity are shown in Table 2 [18, 36]. We used the same set of hypotheses to appraise responsiveness which, in this case, concern a change score of PA [40, 41].

**Table 2:** Cut points for sufficient correlations per construct and dimension of PA measured by the questionnaire, and level of quality

Construct/dimension	1: Very good	2: Adequate	3: Doubtful
Total PAEE (MET/kcal)	Doubly labeled water ≥ 0.70	Accelerometer total counts or average counts ≥ 0.50	Diary, logbook, other questionnaire, interview ≥ 0.70; pedometer steps ≥ 0.40; accelerometer time in moderate, moderate-to-vigorous or vigorous intensity ≥ 0.40
Total PA (min/score)	Accelerometer total counts or average counts ≥ 0.50	Accelerometer time in moderate-to-vigorous intensity ≥ 0.40	Diary, logbook, other questionnaire, interview ≥ 0.70; pedometer steps ≥ 0.40
By intensity			
Vigorous	Accelerometer time in vigorous intensity ≥ 0.50	Accelerometer total counts or average counts ≥ 0.40	Diary, logbook, other questionnaire, interview ≥ 0.70; accelerometer time in light, moderate or moderate-to-vigorous intensity ≥ 0.40; pedometer steps ≥ 0.40
Moderate-to-vigorous	Accelerometer time in moderate-to-vigorous intensity ≥ 0.50	Accelerometer total counts or average counts ≥ 0.40	Diary, logbook, other questionnaire, interview ≥ 0.70; accelerometer time in light, moderate or vigorous intensity ≥ 0.40; pedometer steps ≥ 0.40
Moderate	Accelerometer time in moderate intensity ≥ 0.50	Accelerometer total counts or average counts ≥ 0.40	Diary, logbook, other questionnaire, interview ≥ 0.70; accelerometer time in light, moderate-to-vigorous or vigorous intensity ≥ 0.40; pedometer steps ≥ 0.40
Light	Accelerometer time in light intensity ≥ 0.50	Accelerometer total counts or average counts ≥ 0.40	Diary, logbook, other questionnaire, interview ≥ 0.70; accelerometer time in moderate, moderate-to-vigorous or vigorous intensity ≥ 0.40; pedometer steps ≥ 0.40
By type			
Walking	Pedometer or accelerometer walking total counts ≥ 0.70	Accelerometer total counts or average counts ≥ 0.40	Diary, logbook, other questionnaire, interview ≥ 0.70; accelerometer time in moderate, moderate-to-vigorous or vigorous intensity ≥ 0.40
Leisure time	Accelerometer total counts or average counts in leisure time ≥ 0.50	Accelerometer total counts or average counts ≥ 0.40	Diary, logbook, other questionnaire, interview ≥ 0.70; pedometer steps ≥ 0.40; accelerometer time in moderate, moderate-to-vigorous or vigorous intensity ≥ 0.40
Occupational	Direct observational method ≥ 0.60; accelerometer total counts or average counts during working hours ≥ 0.50	Accelerometer total counts or average counts ≥ 0.40	Diary, logbook, other questionnaire, interview ≥ 0.70; accelerometer time in light, moderate, moderate-to-vigorous or vigorous intensity ≥ 0.40; pedometer steps ≥ 0.40
Household/caregiving	Accelerometer time in light, light-to-moderate or moderate intensity ≥ 0.50	Accelerometer total counts or average counts ≥ 0.40	Diary, logbook, other questionnaire, interview ≥ 0.70; accelerometer time in moderate-to-vigorous or vigorous intensity ≥ 0.40; pedometer steps ≥ 0.40
Sports/exercise	Accelerometer time in moderate-to-vigorous or vigorous intensity ≥ 0.50	Accelerometer total counts or average counts ≥ 0.40	Diary, logbook, other questionnaire, interview ≥ 0.70; accelerometer time in light or moderate intensity ≥ 0.40; pedometer steps ≥ 0.40

*Kcal* kilocalories, *MET* metabolic equivalent, *min* minutes, *PA* physical activity, *PAEE* physical activity energy expenditure

### Quality of Individual Studies

The standards for the assessment of the quality of each study were based on the QAPAQ checklist [36] and were described in our previous reviews [18, 28–30]. Briefly, if the study did not show any substantial flaws in the design or analysis (4: inadequate quality), we assigned one of the three different levels of quality (1: very good, 2: adequate, 3: doubtful) for each construct/subdimension of the questionnaire (e.g., total PA or MVPA) and measurement property (i.e., reliability, measurement error, hypotheses testing for construct validity, and responsiveness).

Reliability and measurement error are usually assessed by repeated measurements in stable participants. To guarantee that the behavior was sufficiently stable over this period [42], we defined an adequate time interval between test and retest as follows: > 1 day and ≤ 3 months for questionnaires recalling a usual week/month; > 1 day and ≤ 2 weeks for questionnaires recalling the previous week; > 1 day and ≤ 1 week for questionnaires recalling the previous day; > 1 day and ≤ 1 year for questionnaires recalling the previous year or assessing lifetime PA. Thus, the following levels of quality for studies on reliability and measurement error were applied:

1. Very good (1): reporting of ICC, LOA, SDC, SEM, CV, kappa or concordance and an adequate time interval between test and retest.
2. Adequate (2): reporting of ICC, LOA, SDC, SEM, CV, kappa or concordance and an inadequate time interval between test and retest; or reporting of Pearson/Spearman correlation and an adequate time interval between test and retest.
3. Doubtful (3): reporting of Pearson/Spearman correlation and an inadequate time interval between test and retest.

Regarding hypotheses testing for construct validity and responsiveness, higher quality was considered with increasing degree of comparability between the measured construct/subdimension and other PA measures (Table 2). For example, the quality was higher for comparisons with accelerometers compared to diaries or other questionnaires.

### Inclusion of the Evidence from the Previous Review

All studies from the previous review [28] were included in this update. Compared to the previous review, the following changes were made within this update: (1) all results were rated irrespective of the sample size. The sample size was considered in the assessment of the quality of the body of evidence; (2) results for measurement error were rated; (3) results based on comparisons with non-PA measures such as health or performance associations were not included; (4) we did not evaluate group differences based on significance levels and instead, only evaluated the magnitude of the effect (e.g., correlation coefficients) [36]; and (5) we used updated levels of quality, as described earlier [18] [e.g., sports/exercise was included in the list, PAEE was distinguished from PA (e.g., as behavior typically measured using raw units such as minutes)]. Due to these differences, two researchers independently (MCS, JJ) reassessed all studies included in the previous review.

### Quality of the Body of Evidence

Based on all studies included from the new and previous review, the quality of evidence was evaluated for the overall construct of each questionnaire (e.g., total PA, total PAEE, total LTPA), also called the ‘total’ score, as well as for the subdimensions MVPA and walking. This was done using the Grading of Recommendation, Assessment, Development, and Evaluation (GRADE) approach [43]. Specifically, we applied a modified approach, as recommended (and described) in the COnsensus-based Standards for the selection of health Measurement INstruments (COSMIN) guideline [33], and assessed the evidence for each measurement property (reliability, measurement error, hypotheses testing for construct validity, and responsiveness) and questionnaire separately. Where applicable, the results from multiple studies on the same questionnaire were summarized. Although different language versions should be treated separately, one may consider summarizing the results if the results have been consistent [33]. Thus, we also assessed the quality of evidence based on the summarized results across multiple studies on different language versions of the same questionnaire.

The grading procedure was described previously [18, 33]. Briefly, the quality of evidence could be high, moderate, low or very low depending on the assessment of four factors (risk of bias (methodological quality of the study), inconsistency in results, indirectness, imprecision). Due to serious flaws in one or more of these factors, the quality of evidence could be downgraded by up to three levels (serious, very serious, extremely serious). For example, serious risk of bias and serious indirectness would result in low-quality evidence (downgraded by two levels).

The assessment of risk of bias was based on the quality ratings of each study (see Sect. 2.5). We considered risk of bias as serious when there were multiple studies of doubtful quality or only one study of adequate quality available, and as very serious when there were multiple studies of inadequate quality or only one study of doubtful quality. We considered downgrading by three levels (extremely serious), if there was only one study of inadequate quality available. Due to inconsistency in results among multiple studies (e.g., some have been sufficient but others insufficient), downgrading by one or two levels was considered. If this inconsistency could be explained, for instance by differences in the study methods (e.g., different subpopulations) or handling of questionnaire data (e.g., score calculation), the results from these studies were not summarized, and the evidence was provided separately. With respect to the purpose of this review (e.g., eligibility criteria), differences in populations and questionnaire scores were evaluated and if applicable, downgrading by one or two levels because of serious or very serious indirectness was considered. For example, one may consider serious indirectness if a study included only male older adults. Finally, imprecision was assessed using the previously determined optimal information sizes for reliability and hypotheses testing for construct validity [18]. If the total sample size did not meet the criteria, we downgraded the evidence by one (serious imprecision, reliability and measurement error: *n* < 45; hypotheses testing for construct validity and responsiveness: *n* < 123) or two (very serious imprecision, reliability and measurement error: *n* < 12; hypotheses testing for construct validity and responsiveness: *n* < 32) levels. Based on the quality of evidence (high, moderate, low, very low) and overall result of the measurement properties (sufficient, insufficient), recommendations for the most-qualified questionnaires were given.

## Results

### Literature Search

**Fig. 1:**
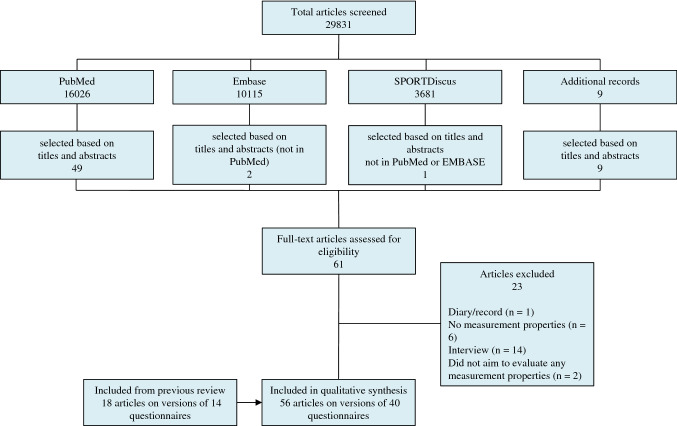
Flow diagram of literature search and study inclusion

The update resulted in 29,831 hits (Fig. 1). Based on titles and abstracts, 61 articles were selected, of which 23 were excluded after reading the full texts. Consequently, 38 articles [44–81] were included in the update. A summary of all included studies, questionnaires and evaluated measurement properties of this update is provided in Table 3.

**Table 3:** Explanation of acronyms or abbreviated names of questionnaires, studies on measurement properties and sample characteristics included in the update

Abbreviation	Full name of questionnaire	Studies on measurement properties	Assessed measurement properties				Comparison measures	Sample *N* (of consented),* n* (women), age (years), BMI (kg/m^2^), specific characteristics, nationality
			Reliability	Measurement error	Hypotheses testing for construct validity	Responsiveness		
AAFQ	Arizona Activity Frequency Questionnaire	Neuhouser et al. [65] English version			●		DLW	450 (of 450), all ♀, age ≥ 60, 31.8% BMI (18.5–24.9), USA
AAS	Active Australia Survey	Vandelanotte et al. [77] English version			●	●	Acc	342 (N/A); 207 ♀, age *n* (%): 50–64 years = 200 (58.8%), age *n* (%): ≥ 65 years = 142 (41.5%), Australia
		Freene et al. [55] English version			●		Acc	First group: 39 (of 56), 29 ♀, mean age = 56.7 (SD = 4.7), mean BMI = 26.9 (SD = 5.1), Australia Second group: 37 (of 40), 26 ♀, mean age = 59.9 (SD = 5.1), mean BMI = 28.1 (SD = 4.7), Australia
		Heesch et al. [58] English version			●		Ped	53 (N/A), 26 ♀, mean age = 72.6 (SD = 5.9), 37.7% BMI (18.5–24.9), 49.1% BMI (25–29.9), 13.2% BMI (≥ 30), Australia
ACLS-PALS	Aerobic Center Longitudinal Study—Physical Activity Long Survey	Banda et al. [46] English version			●		Acc	71 (of 80), 49 ♀, mean age = 57.4 (SD = 9.9), mean BMI = 27.9 (SD = 4.9), 70.5% overweight/obese, 74.6% Caucasian, USA
ACLS-PASS	Aerobic Center Longitudinal Study—Physical Activity Short Survey	Banda et al. [46] English version			●		Acc	71 (of 80), 49 ♀, mean age = 57.4 (SD = 9.9), mean BMI = 27.9 (SD = 4.9), 70.5% overweight/obese, USA
Active-Q	Web-based Physical Activity Questionnaire Active-Q	Bonn et al. [48] Swedish version	●		●		Acc	148 (of 167), all ♂, mean age = 65.4 (SD = 8.7), mean BMI = 25.7 (SD = 2.9), Sweden
BRHS	British Regional Heart Study Physical Activity Questionnaire	Jefferis et al. [62] English version			●		Acc	1377 (of 1655), all ♂, mean age = 78.5 (SD = 4.6), mean BMI = 27.1 (SD = 3.8), UK
Cambridge Index	Simple Physical Activity Index of the European Prospective Investigation into Cancer (EPIC) study	España-Romero et al. [53] English version			●		Acc+HR	1689 (of 1829), 876 ♀, age (range) = 60–64, mean BMI ♀ = 27.9 (SD = 5.3), mean BMI ♂ = 27.8 (SD = 4.2), 32.5% normal-weight ♀, 27.3% normal-weight ♂, UK
CHAMPS	Community Health Activities Model Program for Seniors	Colbert et al. [49] English version	●	●	●		Acc, DLW	56 (of 70), 79% ♀, mean age = 74.7 (SD = 6.5), mean BMI = 25.8 (SD = 4.2), *n* (arthritis) = 50, USA
		Hekler et al. [59] Modified English version	●		●		Acc	870 (25% of 3911), 493 ♀, age ≥ 66, 29.4–56.0% BMI (25–29.9), 13.0–25.2% BMI (≥ 30), USA
EPAQ2	EPIC-Norfolk Physical Activity Questionnaire (based on the EPAQ)	España-Romero et al. [53] Modified English version			●		Acc+HR	1689 (of 1829), 876 ♀, age (range) = 60–64, mean BMI ♀ = 27.9 (SD = 5.3), mean BMI ♂ = 27.8 (SD = 4.2), 32.5% normal-weight ♀, 27.3% normal-weight ♂, UK
GPPAQ	General Practice Physical Activity Questionnaire (based on the Cambridge Index)	Ahmad et al. [44] English version	●		●		Acc	298 (N/A), 160 ♀, age (range) = 60–74, 67% overweight or obese, adults within primary health care, UK
IPAQ-E	International Physical Activity Questionnaire for the Elderly (based on the IPAQ-SF)	Hurtig-Wennlöf et al. [60] Swedish version			●		Acc	54 (of 70), 31 ♀, median age ♀ = 74 (IQR = 69–77), median age ♂ = 71 (IQR = 68–76), Sweden
IPAQ-LF	International Physical Activity Questionnaire—long-form	Cleland et al. [78] English version			●		Acc	226 (of 253), 97 ♀, mean age = 71.8 (SD = 6.6), 81.9% retired, Northern Ireland
		Winckers et al. [74] Modified Dutch version			●		Acc	196 (of 202), 111♀, mean age = 57.1 (SD = 15.4), BMI = 24.8 (SD = 4.2), The Netherlands
		Milanović et al. [64] Serbian version	●					660 (of 700), 308 ♀, mean age = 67.7 (SD = 5.8, mean BMI = 25.9 (SD = 3.7), Serbia
IPAQ-SF	International Physical Activity Questionnaire—short-form	Grimm et al. [56] English version			●		Acc	127 (N/A), 96 ♀, mean age = 63.9 (SD = 7.7), mean BMI = 28.3 (SD = 5.8), USA
		Tomioka et al. [72] Japanese version	●		●		Acc	325 (of 349), 161 ♀, median age ♀(young old) = 70, median age ♂ (young old) = 69, age (range, young old) = 65–74, median age ♀(old old) = 77, median age ♂ (old old) = 78, age (range, old old) = 75–89, 4.8–18.2% BMI (> 25), Japan
		Colpani et al. [50] Portuguese version			●		Ped	292 (of 301), all ♀, mean age = 57.1 (SD = 5.4), mean BMI = 28.3 (SD = 7.0), Brazil
IPEQ	Incidental and Planned Exercise Questionnaire	Delbaere et al. [51] English version	●					500 (N/A), 279 ♀, mean age = 77.4 (SD = 6.08), Australia
LAPAQ	Longitudinal Aging Study Amsterdam Physical Activity Questionnaire	Koolhaas et al. [63] Dutch version			●		Acc	1410 (of 3156), 742 ♀, mean age = 73.8 (SD = 7.6), 70.4% BMI (overweight/obese), The Netherlands
		Siebeling et al. [69] Dutch version	●	●	●		Acc	89 (of 92), 46 ♀, median age = 72.4, age (range) = 65.4–87.6, median BMI = 25.0 BMI (range) = 17.0–35.7, The Netherlands
mLTPA-Q	Modified Leisure Time Physical Activity Questionnaire	Fowles et al. [54] English version	●		●		Acc	32 (of 35), 26 ♀, mean age ♀ = 55 (SD = 10), mean age ♂ = 63 (SD = 9), mean BMI ♀ = 31 (SD = 6), mean BMI ♂ = 26 (SD = 3), Canada
Modified Minnesota LTPA-Q	Modified version of the Minnesota Leisure Time Physical Activity Questionnaire	Sabia et al. [67] English version			●		Acc	3975 (of 4492), 26% ♀, age ≥ 60, UK
MVPA questions	Two questions asking about time spent in Moderate-to-vigorous Physical Activities	Ekblom et al. [35] Swedish version			●		Acc	948 (of 1111), 486 ♀, median age ♀ = 57.5 (IQR = 53.7–61.4), median age ♂ = 57.7 (IQR = 53.8–62.0), Sweden
NC85+PAQ	Newcastle 85 + Study Physical Activity Questionnaire	Innerd et al. [61] English version			●		Acc	484 (N/A), 308 ♀, age (range) = 87–89, 43% BMI (18.5–24.9), UK
NPAQ	Neighborhood Physical Activity Questionnaire	Bödeker et al. [47] German version			●		Ped	58 (of 132), 70.7% ♀, age ≥ 60, Germany
PASB-Q	Physical Activity and Sedentary Behavior Questionnaire (of the Canadian Society for Exercise Physiology)	Fowles et al. [54] English version	●		●		Acc	32 (of 35), 26 ♀, mean age ♀ = 55 (SD = 10), mean age ♂ = 63 (SD = 9), mean BMI ♀ = 31 (SD = 6), mean BMI ♂ = 26 (SD = 3), Canada
PASE	Physical Activity Scale for the Elderly	Ngai et al. ^a^ [66] Chinese version	●					90 (N/A), 54 ♀, mean age = 77.7 (SD = 7.7), mean BMI = 24.4 (SD = 3.8), China
		Vaughan et al.^a^ [73] Chinese version	●	●				73 (N/A), 71% ♀, mean age = 79.0 (SD = 8.5), Chinese immigrants living in Vancouver for at least 5 years, Canada
		Covotta et al.^a^ [79] Italian version	●					94 (of 100), 49.5% ♀, mean age = 62.9 (SD = 7.2), Italy
		Keikavoosi-Arani et al.^a^ [80] Persian version	●					278 (N/A), 65% ♀, mean age = 74.2 (SD = 14.8), mean BMI = 28.2 (SD = 9.9), Iran
		Ayvat et al. [81] Turkish version	●		●		Q	80 (N/A), 29 ♀, mean age = 69.7 (SD = 4.6), mean BMI = 27.7 (SD = 4.9), Turkey
PAVS	Physical Activity Vital Sign Questionnaire	Ball et al. [45] English version			●		Q	298 (of 305), 115 ♀, age *n* (%): ≥ 55 years = 202 (67.8%), adults within primary health care, USA
PHAS question	Public Health Agency of Sweden Physical Activity Question	Ekblom et al. [52] Swedish version			●		Acc	948 (of 1111), 486 ♀, median age ♀ = 57.5 (IQR = 53.7–61.4), median age ♂ = 57.7 (IQR = 53.8–62.0), Sweden
QAPPA	Questionnaire d’Activité Physique pour les Personnes Âgées (Physical Activity Questionnaire for the Elderly)	de Souto Barreto [70] French version	●		●		Q	265 (N/A), 62.9% ♀, mean age = 70.7 (SD = 7.3), France
SBAS	Stanford Brief Activity Survey	Taylor-Piliae et al.^a^ [71] English version	●					1017 (of 1023), 382 ♀, mean age = 65.8 (SD = 2.8), mean BMI = 28.4 (SD = 5.2), USA
SGPALS (LT question)	Saltin-Grimby Physical Activity Level Scale (single question about LTPA)	Ekblom et al. [52] Swedish version			●		Acc	948 (of 1111), 486 ♀, median age ♀ = 57.5 (IQR = 53.7–61.4), median age ♂ = 57.7 (IQR = 53.8–62.0), Sweden
Single item on Recreational and Domestic Activity	Single item on Recreational and Domestic Activity (from the British Regional Heart Study)	Jefferis et al. [62] English version			●		Acc	1377 (of 1655), all ♂, mean age = 78.5 (SD = 4.6), mean BMI = 27.1 (SD = 3.8), UK
Walking question	Single question asking about time spent Walking	Ekblom et al. [52] Swedish version			●		Acc	948 (of 1111), 486 ♀, median age ♀ = 57.5 (IQR = 53.7–61.4), median age ♂ = 57.7 (IQR = 53.8–62.0), Sweden
WHI-PAQ	Women’s Health Initiative Physical Activity Questionnaire	Neuhouser et al. [65] English version			●		DLW	450 (of 450), all ♀, age ≥ 60, 31.8% BMI (18.5–24.9), USA
WHS-AASPA	Women’s Health Study: Accelerometer Ancillary Study Physical Activity Form (based on the NHS II Activity Questionnaire)	Shiroma et al. [68] English version			●		Acc	10,115 (of 16,689), all ♀, mean age = 71.6 (SD = 5.7), mean BMI = 26.1, USA
ZPAQ	Zutphen Physical Activity Questionnaire	Harris et al. [57] English version			●		Acc	234 (of 240), 110 ♀, mean age = 73.6 (SD = 6.1), mean BMI = 27.0 (SD = 4.0), UK
		Harris et al. [57] Modified English version			●		Acc, Ped	234 (of 240), 110 ♀, mean age = 73.6 (SD = 6.1), mean BMI = 27.0 (SD = 4.0), UK

*Acc* accelerometer, *BMI* body mass index, *DLW* doubly labeled water, *EPAQ* Epic Physical Activity Questionnaire, *EPIC* European Prospective Investigation into Cancer, *HR* heart rate, *IQR* interquartile range, *LT* leisure time, *LTPA* leisure time physical activity, *N/A* not applicable, *NHS* Nurses’ Health Study, *PA* physical activity, *Ped* pedometer, *Q* questionnaire, *SD* standard deviation, *UK* United Kingdom, *USA* United States of America

^a^Results for hypotheses testing for construct validity were not included since comparisons were performed with non-PA measures

In the previous review from 2010 [28], 18 articles [82–99] on versions of 13 different questionnaires were included. However, during the reference check of our update, we found two articles [75, 76] which were not included in the previous review. These articles fullfilled all our inclusion criteria, have been published before September 2008, and, thus, were now included. Results from studies reported in these two articles were shown together with those from previously included studies in order to allow comparisons. An overview of all previously included studies (including the latter two articles) is provided in Electronic Supplementary Material Table S1. In contrast to 2010, we considered the Cambridge Index as a stand-alone instrument which means that we reassessed 14 (instead of 13) different questionnaires. Six questionnaires [Cambridge Index, Community Health Activities Model Program for Senior (CHAMPS), International Physical Activity Questionnaire—short-form (IPAQ-SF), PASE, Stanford Brief Activity Survey (SBAS), Women’s Health Initiative Physical Activity Questionnaire (WHI-PAQ)] were assessed in studies included both in the update and previous review.

Previous review and update combined, we included studies on measurement properties of versions of 40 different questionnaires (14 from the previous review and 26 from the update) derived from 56 articles. Information about reliability was available for versions of 22, measurement error for four, and hypotheses testing for construct validity for 38 different questionnaires. Results for responsiveness were available for one questionnaire. Regarding the latter measurement property, one study [100] from the update was excluded after reading the full text because the reported results for responsiveness could not be evaluated with respect to our set of hypotheses. Likewise, another study [82] from the previous review evaluated the sensitivity to change of the CHAMPS but did not use a PA comparison measure or test hypotheses about expected effect sizes.

Three studies [49, 65, 83] considered doubly labeled water (DLW) as a comparison method, whereas most often accelerometers, pedometers and other PA questionnaires were used. Both original and modified versions were assessed. For example, two studies modified the CHAMPS by replacing questions and adjusting MET values [59] or changing the recall period to the past 7 days (instead of past 4 weeks) and using modified response categories [84]. Some studies evaluated measurement properties of new indices [e.g., Cambridge Index derived from the questionnaire used in the European Prospective Investigation into Cancer and Nutrition (EPIC)].

Finally, although all studies evaluated a ‘PA questionnaire’, two studies evaluated questionnaires intending to measure the construct total EE (i.e., Questionnaire d’Activité Physique Saint-Etienne (QAPSE) [85], Questionnaire preceding EPIC (Pre-EPIC) [86]) and one study presented multiple results concerning both total EE and PA (i.e., Flemish Physical Activity Computerized Questionnaire (FPACQ) [87]). The construct total EE is different from PA, since it also includes a detailed assessment of all activities summing up to 24 h (e.g., rest, sleep, eating). Whenever reported, results for total EE were not evaluated but included in the tables to allow the reader to interpret the results.

### Description of Questionnaires

A detailed description of all questionnaires included in the update is provided in the Electronic Supplementary Material Table S2 whereas a description of previously included questionnaires was provided in 2010 [28]. The populations for which the questionnaires were developed varied (e.g., older adults, female adults). Most questionnaires intend to measure total PA, total PAEE, MVPA or domain-specific PA such as LTPA. Some questionnaires [e.g., Web-based Physical Activity Questionnaire Active-Q (Active-Q)] measure frequency and duration of activities but not the relative intensity in which these activities were performed (i.e., subjective rating of the participants). Although intensity may not be measured in this way, usually absolute MET values were assigned to activities to obtain time spent in different intensity levels (e.g., light, moderate, vigorous). Finally, sometimes information about parameters of PA (frequency, duration, intensity) is only obtained for some but not all listed activities [e.g., Arizona Activity Frequency Questionnaire (AAFQ)].

### Assessment of Measurement Properties

#### Content Validity

Based on our three criteria, the content validity was sufficient for 22 questionnaires [AAFQ, Active Australia Survey (AAS), Aerobic Center Longitudinal Study—Physical Activity Long Survey (ACLS-PALS), Active-Q, CHAMPS, EPIC-Norfolk Physical Activity Questionnaire (EPAQ2), FPACQ, International Physical Activity Questionnaire for the Elderly (IPAQ-E), International Physical Activity Questionnaire—long form (IPAQ-LF), IPAQ-SF, Modified Leisure Time Physical Activity Questionnaire (mLTPA-Q), Modified version of the Minnesota Leisure Time Physical Activity Questionnaire (Modified Minnesota LTPA-Q), Older Adult Exercise Status Inventory (OA-ESI), PASE, Physical Activity and Sedentary Behavior Questionnaire (PASB-Q), Physical Activity Questionnaire for Elderly Japanese (PAQ-EJ), Physical Activity Vital Sign Questionnaire (PAVS), Physical Activity Questionnaire for the Elderly (QAPPA), Pre-EPIC, Two questions asking about time spent in Moderate-to-vigorous Physical Activities (MVPA questions), Walking question, Zutphen Physical Activity Questionnaire (ZPAQ)].

It should be noted that the content validity of the original version of the ZPAQ was insufficient due to the lack of household-related activities [101]. However, the content validity of the modified version of the ZPAQ was sufficient because the authors included the missing domain [57].

#### Reliability and Measurement Error

Table 4 summarizes the results for reliability and measurement error of studies included in the update. The results of the reassessment of all studies included in the previous review are shown in Electronic Supplementary Material Table S3. The quality of studies was usually very good or adequate. Versions of the CHAMPS (English version, Modified English version), IPAQ-SF (Chinese version, Japanese version), OA-ESI (English version), PASE (Chinese version, English version, Italian version, Japanese version, Norwegian version, Persian version, Turkish version) and the Self-administered Physical Activity Questionnaire (Self-administered PAQ; Swedish version) were evaluated in multiple studies.

**Table 4:** Reliability and measurement error of PA questionnaires for older adults

Questionnaire	Study population (*n*) for analysis	Interval	Results	Study quality and result rating^a^
Active-Q Swedish version Bonn et al. [48]	148	3 weeks	Light: ICC = 0.66 [0.57–0.75]	1−
			Moderate: ICC = 0.69 [0.60–0.77]	1−
			Vigorous: ICC = 0.51 [0.39–0.63]	1−
			Moderate-to-vigorous: ICC = 0.67 [0.58–0.76]	1−
			Sedentary-to-light: ICC = 0.67 [0.58–0.76]	
			Sedentary: ICC = 0.80 [0.74–0.86]	
CHAMPS English version Colbert et al. [49]	56	10 days	Total (PAEE): ICC = 0.64	1−
			Measurement error:	
			Total (PAEE): $\bar{d}$ = − 11, LOA^b^ = − 11 ± 1.96*181 (kcal/day)	1−
CHAMPS Modified English version Hekler et al. [59]	748	6 months	Total (duration): ICC = 0.69	2−
			Total (PAEE): ICC = 0.64	2−
			Low-light (duration): ICC = 0.70	2+
			High-light (duration): ICC = 0.68	2−
			Moderate-to-vigorous (duration): ICC = 0.66	2−
			Moderate-to-vigorous (PAEE): ICC = 0.61	2−
			Sedentary (duration): ICC = 0.56	
GPPAQ English version Ahmad et al. [44]	126	3 months	Total: *κ* = 0.57	1−
	129	12 months	Total: *κ* = 0.63	2−
IPAQ-LF Serbian version Milanović et al. [64]	660 (*n*_men_ = 352, *n*_women_ = 308)	2 weeks	Total (PAEE): ICC_men_ = 0.71 [0.58–0.82]; ICC_women_ = 0.74 [0.59–0.83]	1+ 1+
			Moderate: ICC_men_ = 0.77 [0.71–0.87]; ICC_women_ = 0.64 [0.53–0.69]	1+ 1−
			Vigorous: ICC_men_ = 0.88 [0.79–0.94]; ICC_women_ = 0.82 [0.75–0.89]	1+ 1+
			Walking: ICC_men_ = 0.69 [0.55–0.81]; ICC_women_ = 0.61 [0.58–0.72]	1− 1−
			Work: ICC_men_ = 0.64 [0.51–0.71]; ICC_women_ = 0.85 [0.79–0.93]	1− 1+
			Transport: ICC_men_ = 0.71 [0.62–0.79]; ICC_women_ = 0.91 [0.81–0.96]	1+ 1+
			Housework/gardening: ICC_men_ = 0.68 [0.56–0.75]; ICC_women_ = 0.90 [0.80–0.95]	1− 1+
			Leisure: ICC_men_ = 0.53 [0.42–0.64]; ICC_women_ = 0.74 [0.68–0.81]	1− 1+
IPAQ-SF Japanese version Tomioka et al. [72]	325 (*n*_women+aged 65–74_ = 88; *n*_men+aged 65–74_ = 81; *n*_women+aged 75–89_ = 73; *n*_men+aged 75–89_ = 83)	2 weeks	Total (PAEE; age group: 65–74): ICC_men_ = 0.65 [0.46–0.78]; ICC_women_ = 0.57 [0.34–0.72]	1− 1−
			Total (PAEE; age group: 75–89): ICC_men_ = 0.50 [0.22–0.68]; ICC_women_ = 0.56 [0.30–0.72]	1− 1−
			Moderate (age group: 65–74): ICC_men_ = 0.52 [0.25–0.69]; ICC_women_ = 0.47 [0.18–0.65]	1− 1−
			Moderate (age group: 75–89): ICC_men_ = 0.63 [0.43–0.76]; ICC_women_ = 0.60 [0.36–0.75]	1− 1−
			Vigorous (age group: 65–74): ICC_men_ = 0.55 [0.31–0.71]; ICC_women_ = 0.58 [0.36–0.73]	1− 1−
			Vigorous (age group: 75–89): ICC_men_ = 0.39 [0.06–0.61]; ICC_women_ = 0.30 [-0.11–0.56]	1− 1−
			Walking (age group: 65–74): ICC_men_ = 0.73 [0.59–0.83]; ICC_women_ = 0.55 [0.32–0.71]	1+ 1−
			Walking (age group: 75–89): ICC_men_ = 0.65 [0.46–0.77]; ICC_women_ = 0.60 [0.36–0.75]	1− 1−
			Sitting (age group: 65–74): ICC_men_ = 0.82 [0.71–0.88]; ICC_women_ = 0.70 [0.54–0.80]	
			Sitting (age group: 75–89): ICC_men_ = 0.66 [0.48–0.78]; ICC_women_ = 0.67 [0.48–0.80]	
IPEQ English version Delbaere et al. [51]	*n*_past week version_ = 30; *n*_past 3 months version_ = 50	1 week	Total (last week version): ICC = 0.77	1+
			Total (last 3 months version): ICC = 0.84	1+
LAPAQ Dutch version Siebeling et al. [69]	86 (*n*_representative sample_ = 50)	2 weeks	Total (overall sample): *r* = 0.68 [0.55–0.80]	2−
			Total (representative sample): *r* = 0.73 [0.59–0.88]	2−
			Mild (overall sample): *r* = 0.58 [0.42–0.72]	2−
			Mild (representative sample): *r* = 0.69 [0.54–0.84]	2−
			Moderate (overall sample): *r* = 0.79 [0.69–0.88]	2−
			Moderate (representative sample): *r* = 0.81 [0.69–0.93]	2+
			Vigorous (overall sample): *r* = 0.75 [0.47–0.87]	2−
			Vigorous (representative sample): *r* = 0.81 [0.49–0.93]	2+
			Measurement error:	
			Total: $\bar{d}$ = 436, LOA^b^ = 436 ± 1.96*1260 (min/2 weeks)	1−
			Mild: $\bar{d}$ = 309, LOA^b^ = 309 ± 1.96*1004 (min/2 weeks)	1−
			Moderate: $\bar{d}$ = 102, LOA^b^ = 102 ± 1.96*436 (min/2 weeks)	1−
			Vigorous: $\bar{d}$ = 23, LOA^b^ = 23 ± 1.96*258 (min/2 weeks)	1−
mLTPA-Q English version Fowles et al. [54]	35	1 week	Mild (LTPA): *r* = 0.04	2−
			Moderate (LTPA): *r* = 0.49	2−
			Strenuous (LTPA): *r* = 0.45	2−
			Moderate-to-vigorous (LTPA): *r* = 0.66	2−
PASB-Q English version Fowles et al. [54]	35	1 week	Moderate-to-vigorous (PAVS): *r* = 0.83	2+
			Muscle-strengthening (frequency): *r* = 0.92	2+
PASE Chinese version Ngai et al. [66]	32	N/A	Total: ICC = 0.81	? +
PASE Chinese version Vaughan et al. [73]	66	2 weeks	Total: ICC = 0.79 [0.68–0.86]	1+
			Walking outside home: *κ* = 0.45	1−
			Light sports/recreational activities: *κ* = 0.33	1−
			Moderate sports/recreational activities: *κ* = 0.51	1−
			Strenuous sports/recreational activities: *κ* = 0.65	1−
			Muscle strength/endurance exercise: *κ* = 0.43	1−
			Light housework: *κ* = 0.78	1+
			Heavy housework or chores: *κ* = 0.64	1−
			Home repairs: *κ* = 0.39	1−
			Lawn work or yard care: *κ* = 0.17	1−
			Outdoor gardening: *κ* = 0.85	1+
			Caring for another person: *κ* = 0.62	1−
			Work for pay or as a volunteer: *κ* = 0.92	1+
			Measurement error:	
			Total: MDD_95_ = 63.1, SEM = 22.8 (weighted total score)	1−
			Total: $\bar{d}$ = 2.4, LOA = 2.4 ± 68.5 (weighted total score)	1−
PASE Italian version Covotta et al. [79]	48	1 week	Total: ICC = 0.98 (0.96–0.99)	1+
			Leisure time activity: ICC = 0.99 (0.99–0.99)	1+
			Household activity: ICC = 0.99 (0.98–0.99)	1+
			Work-related activity: ICC = 0.97 (0.94–0.98)	1+
PASE Persian version Keikavoosi-Arani et al. [80]	278	2 weeks	Walking outside home: ICC = 0.90 (0.92–0.94)	1+
			Light sports/recreational activities: ICC = 0.89 (0.87–0.91)	1+
			Moderate sports/recreational activities: ICC = 0.93 (0.90–0.95)	1+
			Strenuous sports/recreational activities: ICC = 0.91 (0.89–0.92)	1+
			Muscle strength/endurance exercise: ICC = 0.92 (0.90–0.95)	1+
			Household activity: ICC = 0.86 (0.82–0.87)	1+
			Light housework: ICC = 0.86 (0.82–0.86)	1+
			Heavy housework or chores: ICC = 0.81 (0.80–0.84)	1+
			Home repairs: ICC = 0.76 (0.72–0.77)	1+
			Lawn work or yard care: ICC = 0.80 (0.79–0.81)	1+
			Caring for another person: ICC = 0.95 (0.92–0.97)	1+
			Job—standing or walking: ICC = 0.91 (0.90–0.94)	1+
PASE Turkish version Ayvat et al. [81]	80	1 week	Total: ICC = 0.99 (0.99–0.99)	1+
			Leisure time activity: ICC = 0.99 (0.99–0.99)	1+
			Household activity: ICC = 0.99 (0.99–0.99)	1+
			Work-related activity: ICC = 1.00 (1.00–1.00)	1+
QAPPA French version de Souto Barreto [70]	225	1 year	Moderate (PAEE): ICC = 0.46	2−
			Vigorous (PAEE): ICC = 0.63	2−
			Moderate-to-vigorous (PAEE): ICC = 0.64	2−
			Classification (active/inactive): *κ* = 0.44	
SBAS Taylor-Piliae et al. [71] English version	996	2 years	Total: *ρ* = 0.62	3−

*Active-Q* Web-based Physical Activity Questionnaire Active-Q*, CHAMPS* Community Health Activities Model Program for Seniors, $\bar{d}$ change in the mean, *GPPAQ* General Practice Physical Activity Questionnaire, *ICC* intraclass correlation coefficient, *κ* Kappa coefficient; *IPAQ-LF* International Physical Activity Questionnaire—long-form, *IPAQ-SF* International Physical Activity Questionnaire—short-form, *IPEQ* Incidental and Planned Exercise Questionnaire, *kcal* kilocalories, *LAPAQ* Longitudinal Aging Study Amsterdam Physical Activity Questionnaire, *LOA* limits of agreement, *LTPA* leisure time physical activity; *MDD*_*95*_ minimal detectable difference based on the 95% confidence interval, *min* minutes, *mLTPA-Q* Modified Leisure Time Physical Activity Questionnaire, *N/A* not applicable, *PA* physical activity, *PAEE* physical activity energy expenditure, *PASB-Q* Physical Activity and Sedentary Behavior Questionnaire, *PASE* Physical Activity Scale for the Elderly, *PAVS* physical activity vital sign, QAPPA Questionnaire d’Activité Physique pour les Personnes Âgées (Physical Activity Questionnaire for the Elderly), *r* Pearson correlation coefficient, *ρ* Spearman correlation coefficient, *SBAS* Stanford Brief Activity Survey, *SEM* standard error of measurement, *?* unclear

^a^As described in Sect. 2.5, the quality of the individual study was evaluated per questionnaire and construct/dimension of PA and can be either very good (1), adequate (2), doubtful (3) or inadequate (4). Additionally, the reported results were rated [i.e., sufficient (+), insufficient (−)] as described in Sect. 2.4

^b^Based on the reported results, we calculated the LOA using the formula LOA = $\bar{d}$ ± 1.96*s*$\surd 2$, where s = within-subject standard deviation (typical error) [146]

In at least one study, versions of 10 questionnaires [CHAMPS, FPACQ, IPAQ-LF, IPAQ-SF, Incidental and Planned Exercise Questionnaire (IPEQ), Modified Baecke, PASB-Q, PASE, QAPSE, WHI-PAQ] showed sufficient reliability in assessing the overall construct (e.g., total PA, total LTPA) and/or subdimensions (i.e., MVPA, walking) of PA. Measurement error was assessed for versions of four questionnaires [CHAMPS, Longitudinal Aging Study Amsterdam Physical Activity Questionnaire (LAPAQ), PASE, Questionnaire used in the EPIC (EPIC)]. The measurement errors of these versions were insufficient for all scores.

#### Construct Validity and Responsiveness

Table 5 shows the results for different hypotheses for construct validity and responsiveness of studies included in this update. The results of the reassessment of all studies included in the previous review are shown in Electronic Supplementary Material Table S4. The level of quality varied but most studies were of very good or adequate quality. Versions of the AAS (English version), Cambridge Index (English version), CHAMPS (English version, Modified English version), IPAQ-LF (English version, Modified Dutch version), IPAQ-SF (Chinese version, English version, Japanese version, Portuguese version), LAPAQ (Dutch version), PASE (Dutch version, English version, Japanese version, Turkish version) and the Self-Administered PAQ (Swedish version) were evaluated in multiple studies.

**Table 5:** Hypotheses testing for construct validity and responsiveness of PA questionnaires for older adults

Questionnaire	Study population (*n*) for analysis	Comparison measure (type, placement, registration period [valid week], epoch length, cut points)	Results	Study quality and result rating^a^
AAFQ English version Neuhouser et al. [65]	450	DLW	Total (PAEE): *R*^2^ = 7.6% (24.0% when corrected for measurement error)	1−
AAS English version Vandelanotte et al. [77]	*n*_50–64 years of age_ = 186, *n*_over 65 years of age_ = 132	Accelerometer (ActiGraph GT3X, hip, waking hours of 7 days [5 days], 1 s, Freedson et al. [136]	*50–64* *years of age*	
			Moderate: *ρ* = 0.24 [0.10–0.38]	1−
			Vigorous: *ρ* = 0.41 [0.27–0.54]	1−
			Moderate-to-vigorous: *ρ* = 0.28 [0.15–0.43]	1−
			*Over 65* *years of age*	
			Moderate: *ρ* = 0.20 [0.02–0.37]	1−
			Vigorous: *ρ* = 0.20 [0.02–0.38]	1−
			Moderate-to-vigorous: *ρ* = 0.21 [0.05–0.38]	1−
	*n*_50–64 years of age_ = 134, *n*_over 65 years of age_ = 104	Accelerometer (ActiGraph GT3X, hip, waking hours of 7 days [5 days], 1 s, Freedson et al. [136]	Responsiveness:	
			*50–64* *years of age*	
			Moderate: *ρ* = 0.36 [0.19–0.51]	1−
			Vigorous: *ρ* = 0.12 [− 0.07 to 0.30]	1−
			Moderate-to-vigorous: *ρ* = 0.36 [0.20–0.51]	1−
			*Over 65* *years of age*	
			Moderate: *ρ* = 0.32 [0.12–0.50]	1−
			Vigorous: *ρ* = 0.31 [0.13–0.47]	1−
			Moderate-to-vigorous: *ρ* = 0.34 [0.13–0.51]	1−
AAS English version Freene et al. [55]	*n*_first group_ = 39, *n*_second group_ = 37	Accelerometer (ActiGraph GT1M, hip, waking hours of 7 days [4 days], 5 s, Freedson et al. [136])	*No minimum bout length*	
			Total (LTPA)^b^: *ρ* = 0.56; *ρ* = 0.49	3+ 3+
			Moderate (LTPA, including walking)^b^: *ρ* = 0.56; *ρ* = 0.55	3+ 3+
			Vigorous (LTPA)^b^: *ρ* = 0.33; *ρ* = − 0.08	3− 3−
			Classification of LTPA (active/inactive)^b^: *φ* = 0.41; *φ* = 0.16	
			*10-min minimum bout length*	
			Total (LTPA)^b^: *ρ* = 0.56; *ρ* = 0.64	3+ 3+
			Moderate (LTPA, including walking)^b^: *ρ* = 0.63; *ρ* = 0.64	3+ 3+
			Vigorous (LTPA)^c^: *ρ* = 0.17	3−
			Classification of LTPA (active/inactive)^b^: *φ* = 0.47; *φ* = 0.21	
AAS English version Heesch et al. [58]	50	Pedometer (Yamax SW-200, waking hours of 7 days [4 days])	Total (LTPA): *ρ* = 0.42	3+
			Moderate-to-vigorous (LTPA, excluding walking): *ρ* = 0.31	3−
			Walking (LTPA): *ρ* = 0.42	1−
ACLS-PALS English version Banda et al. [46]	71	Accelerometer (Actical, waist, 7 days [4 days], 60 s, Hooker et al. [137])	*1-min minimum bout length*	
			Total (Exercise): *r* = 0.55 [0.35–0.75]	1+
			Classification (active/inactive): *κ* = 0.38 [0.16–0.60]	
			*10-min minimum bout length*	
			Total (Exercise): *r* = 0.49 [0.29–0.70]	1−
			Classification (active/inactive): *κ* = 0.15 [0.03–0.28]	
ACLS-PASS English version Banda et al. [46]	71	Accelerometer (Actical, waist, 7 days [4 days], 60 s, Hooker et al. [137])	*1-min minimum bout length*	
			Moderate-to-vigorous: *r* = 0.53 [0.32–0.73]	1+
			Classification (active/inactive): *κ* = 0.26 [0.04–0.48]	
			*10-min minimum bout length*	
			Moderate-to-vigorous: *r* = 0.37 [0.15–0.60]	1−
			Classification (active/inactive): *κ* = 0.04 [ − 0.04 to 0.11]	
Active-Q Swedish version Bonn et al. [48]	148	Accelerometer (GENEA, wrist, two times for 7 days [6 days/week], 60 s, Bonn et al. [48])	Light: *ρ* = 0.15 [0.00–0.31]	1−
			Moderate: *ρ* = 0.27 [0.12–0.42]	1−
			Vigorous: *ρ* = 0.54 [0.42–0.67]	1+
			Moderate-to-vigorous: *ρ* = 0.35 [0.21–0.48]	1−
			Sedentary-to-light: *ρ* = 0.35 [0.19–0.51]	
			Sedentary: *ρ* = 0.19 [0.04–0.34]	
			Moderate (classification based on quartiles): *κ* = 0.16	
			Vigorous (classification based on quartiles): *κ* = 0.39	
			Moderate-to-vigorous (classification based on quartiles): *κ* = 0.22	
			Light: $\bar{d}$ = 87, LOA = − 398 to 571 (min/day)	
			Moderate: $\bar{d}$ = 76, LOA = − 157 to 309 (min/day)	
			Vigorous: $\bar{d}$ = 15, LOA = − 33 to 64 (min/day)	
			Moderate-to-vigorous: $\bar{d}$ = 91, LOA = − 147 to 329 (min/day)	
			Sedentary-to-light: $\bar{d}$ = − 91, LOA = − 329 to 146 (min/day)	
			Sedentary: $\bar{d}$ = − 178, LOA = − 606 to 250 (min/day)	
BRHS English version Jefferis et al. [62]	1377	Accelerometer (Actigraph, GT3X, hip, waking hours of 7 days [3 days], Copeland et al. [138])	Total (compared to cpm; steps; MVPA): *ρ* = 0.49; *ρ* = 0.49; *ρ* = 0.49	1− 3+ 2+
Cambridge Index English version España-Romero et al. [53]	1689	Accelerometer + heart rate (Actiheart/Red Dot 2570: 3M, 5 days [48 h], 30 s, individual/group calibration)	*Women*	
			Total (compared to MVPA; PAEE): *ρ* = 0.21; *ρ* = 0.17	2− 1−
			*Men*	
			Total (compared to MVPA; PAEE): *ρ* = 0.24; *ρ* = 0.25	2− 1−
CHAMPS English version Colbert et al. [49]	56	Accelerometer (ActiGraph GT1M, waist, waking hours of 10 days [≥ 10 h for any day], 10 s, Crouter et al. [139])	Total (PAEE): *ρ* = 0.52	2+
	56	DLW	Total (PAEE): *ρ* = 0.28	1−
			Total (PAI): *ρ* = 0.23	1−
			Total (PAEE): LOA^d^ = − 968 to 130 (− 419 ± 1.96*280 kcal/day)	
CHAMPS Modified English version Hekler et al. [59]	850	Accelerometer (Actigraph models 7164 and 71256, waist, waking hours of 7 days [5 days], 60 s, Freedson et al. [136], Copeland et al. [138])	Total (duration): *ρ* = 0.38	1−
			Total (PAEE): *ρ* = 0.39	2−
			Low-light (duration): *ρ* = 0.06	1−
			High-light (duration): *ρ* = 0.27	1−
			Moderate-to-vigorous (duration): *ρ* = 0.37	1−
			Moderate-to-vigorous (PAEE): *ρ* = 0.38	1−
			Sedentary (duration): *ρ* = 0.12	
			Total (duration): $\bar{d}$ = 618, LOA = − 504 to 1740 (min/week)	
			Low-light (duration): $\bar{d}$ = − 473, LOA = − 1937 to 992 (min/week)	
			High-light (duration): $\bar{d}$ = 396, LOA = − 346 to 1137 (min/week)	
			Moderate-to-vigorous (duration): $\bar{d}$ = 222, LOA = − 403 to 848 (min/week)	
EPAQ2 Modified English version España-Romero et al. [53]	1689	Accelerometer + heart rate (Actiheart/Red Dot 2570: 3M, 5 days [48 h], 30 s, individual/group calibration)	*Women*	
			Total (PAEE)^e^: *ρ* = 0.26	1−
			Light: *ρ* = 0.12	1−
			Moderate-to-vigorous: *ρ* = 0.36	1−
			Sedentary: *ρ* = 0.18	
			Total (PAEE)^e^: $\bar{d}$ = 29, LOA = − 39 to 95 (kJ/kg/day)	
			Light: $\bar{d}$ = − 60, LOA = − 368 to 247 (min/day)	
			Moderate-to-vigorous: $\bar{d}$ = 55, LOA = − 117 to 228 (min/day)	
			Sedentary: $\bar{d}$ = − 6.0, LOA = − 10.9 to 1.0 (h/day)	
			*Men*	
			Total (PAEE)^e^: *ρ* = 0.27	1−
			Light: *ρ* = 0.15	1−
			Moderate-to-vigorous: *ρ* = 0.30	1−
			Sedentary: *ρ* = 0.17	
			Total (PAEE): $\bar{d}$ = 32, LOA = − 62 to 123 (kJ/kg/day)	
			Light: $\bar{d}$ = – 172, LOA = − 455 to 111 (min/day)	
			Moderate-to-vigorous: $\bar{d}$ = 91, LOA = − 160 to 342 (min/day)	
			Sedentary: $\bar{d}$ = − 4.6, LOA = − 10.6 to 1.3 (h/day)	
GPPAQ English version Ahmad et al. [44]	289	Accelerometer (Actigraph, GT3X+, waist, waking hours of 7 days [5 days], 5 s, Freedson et al. [136])	Classification (active/inactive): Sensitivity = 19%	Not rated
			Classification (active/inactive): Specificity = 85%	
			Classification (active/inactive; including walking): Sensitivity = 39%	
			Classification (active/inactive; including walking): Specificity = 70%	
IPAQ-E Swedish version Hurtig-Wennlöf et al. [60]	54	Accelerometer (Actigraph GT1M, waist, waking hours of 7 days [4 days], 15 s, Matthews et al.[140], Swartz et al. [141], Troiano et al. [142])	Moderate^f^: *ρ* = 0.47; *ρ* = 0.17; *ρ* = 0.40	1− 1− 1−
			Moderate plus walking^f^: *ρ* = 0.42, *ρ* = 0.34, *ρ* = 0.41	1− 1− 1−
			Vigorous: *ρ* = 0.37	1−
			Walking: *ρ* = 0.30	2−
			Sitting: *ρ* = 0.28	
			Classification (active/inactive): *κ* = 0.45 [0.18–0.72], Sensitivity = 81%, Specificity = 85%	
IPAQ-LF English version Cleland et al. [78]	226	Accelerometer (ActiGraph GT3X+, waist, waking hours of 7 days [5 days], 15 s, Copeland et al. [138])	Moderate-to-vigorous: *ρ* = 0.52	1+
			Moderate-to-vigorous: $\bar{d}$ = − 100, LOA = 1766 to − 1965 (min/week)	
IPAQ-LF Modified Dutch version Winckers et al. [74]	196	Accelerometer (ActiGraph GT3X+, hip, waking hours of 7 days [5 days])	Total (PAEE): *ρ* = 0.16	3−
			Moderate-to-vigorous (compared with Actigraph total counts): *ρ* = 0.27	2−
			Moderate-to-vigorous (compared with Actigraph MVPA): *ρ* = 0.16	1−
IPAQ-SF English version Grimm et al. [56]	127	Accelerometer (ActiGraph 7164, hip, waking hours of 7 days [5 days], 60 s, Crouter et al. [139], Freedson et al. [136], Matthews et al. [140])	*No minimum bout length:*	
			Total (PAEE): *ρ* = 0.24	3−
			Moderate: *ρ* = 0.16	1−
			Vigorous: *ρ* = 0.18	1−
			Walking: *ρ* = 0.29	3−
			Sitting: *ρ* = 0.36	
			Classification (active/passive): *κ* = 0.02	
			*10-min minimum bout length:*	
			Total (PAEE): *ρ* = 0.17	3−
			Moderate: *ρ* = 0.04	1−
			Vigorous: *ρ* = 0.17	1−
			Walking: *ρ* = 0.28	3−
			Classification (active/passive): *κ* = 0.10	
			Moderate lifestyle: $\bar{d}$ = − 15, LOA = − 214 to 184 (min/day)	
			Moderate walking: $\bar{d}$ = − 27, LOA = − 143 to 90 (min/day)	
			Vigorous: $\bar{d}$ = − 43, LOA = − 176 to 90 (min/day)	
			Sitting: $\bar{d}$ = 262, LOA = − 84 to 608 (min/day)	
IPAQ-SF Japanese version Tomioka et al. [72]	306 (*n*_women+aged 65–74_ = 84; *n*_men+aged 65–74_ = 76; *n*_women+aged 75–89_ = 69; *n*_men+aged 75–89_ = 77)	Accelerometer (Kenz Lifecorder PLUS, waist, waking hours of at least 28 days [14 days], Tomioka et al. [72])	Total (PAEE; age group: 65–74): *ρ*_men_ = 0.42, *ρ*_women_ = 0.49	2− 2−
			Total (PAEE; age group: 75–89): *ρ*_men_ = 0.53, *ρ*_women_ = 0.49	2+ 2−
			Moderate (age group: 65–74): *ρ*_men_ = 0.26, *ρ*_women_ = 0.13	2− 2−
			Moderate (age group: 75–89): *ρ*_men_ = 0.05, *ρ*_women_ = 0.03	2− 2−
			Vigorous (age group: 65–74): *ρ*_men_ = 0.25, *ρ*_women_ = 0.12	2− 2−
			Vigorous (age group: 75–89): *ρ*_men_ = 0.17, *ρ*_women_ = 0.17	2− 2−
			Walking (age group: 65–74): *ρ*_men_ = 0.30, *ρ*_women_ = 0.48	1− 1−
			Walking (age group: 75–89): *ρ*_men_ = 0.59, *ρ*_women_ = 0.55	1− 1−
			Classification (tertiles; age group: 65–74): *κ*_men_ = 0.50 [0.36–0.64], *κ*_women_ = 0.39 [0.22–0.56]	
			Classification (tertiles; age group: 75–89): *κ*_men_ = 0.47 [0.31–0.63], *κ*_women_ = 0.47 [0.28–0.66]	
IPAQ-SF Portuguese version Colpani et al. [50]	292	Pedometer (BP 148 TECHLINE, waist, waking hours of 7 days)	Total (PAEE): *ρ* = 0.11	3−
			Classification (active/inactive): *κ* = 0.11	
			Classification (active/moderate/inactive): *κ* = 0.08	
			Total: $\bar{d}$ = − 0.17, LOA = − 2.36 to 2.03 (*Z* scores of steps and MET_log_)	
LAPAQ Dutch version Koolhaas et al. [63]	1410	Accelerometer (GeneActiv, wrist, 7 days [4 days], White et al. [143])	Total: *ρ* = 0.30 [0.25–0.34]	1−
			Total: $\bar{d}$ = – 529, LOA^d^ = − 529 ± 1.96*1080 (min/week)	
			Light: $\bar{d}$ = − 708, LOA^d^ = − 708 ± 1.96*484 (min/week)	
			Moderate: $\bar{d}$ = 205, LOA^d^ = 205 ± 1.96*781 (min/week)	
			Vigorous: $\bar{d}$ = – 26, LOA^d^ = − 26 ± 1.96*338 (min/week)	
LAPAQ Dutch version Siebeling et al. [69]	88	Accelerometer (Sensewear Pro, upper arm, 14 days)	Total: *r* = 0.25 [0.07–0.44]	1−
			Mild: *r* = 0.05 [ − 0.16 to 0.24]	1−
			Moderate: *r* = 0.27 [0.07–0.48]	1−
			Vigorous: *r* = 0.01 [− 0.07 to 0.25]	1−
			Classification (active/inactive): AUC: 0.73 [0.59–0.86]	
			Total: $\bar{d}$ = − 354, LOA^d^ = − 354 ± 1.96*1830 (min/2 weeks)	
			Mild: $\bar{d}$ = − 267, LOA^d^ = − 267 ± 1.96*1423 (min/2 weeks)	
			Moderate: $\bar{d}$ = − 234, LOA^d^ = − 234 ± 1.96*852 (min/2 weeks)	
			Vigorous: $\bar{d}$ = 148, LOA^d^ = 148 ± 1.96*403 (min/2 weeks)	
mLTPA-Q English version Fowles et al. [54]	32	Accelerometer (ActiGraph GT3X, hip, waking hours of 7 days [4 days], Freedson et al. [136])	Moderate (LTPA): *r* = 0.53	3+
			Strenuous (LTPA): *r* = 0.18	3−
			Moderate-to-vigorous (LTPA): *r* = 0.56	3+
			Classification (active/inactive): Sensitivity = 73%, Specificity = 82%	
			Moderate-to-vigorous (LTPA): LOA = − 223 to 262 (min/week)	
Modified Minnesota LTPA-Q English version Sabia et al. [67]	3975	Accelerometer (GeneActiv. Wrist, 9 days [4 days])	Total (PAEE): *ρ* = 0.33 [0.30–0.36]	2–
			Mild: *ρ* = 0.21 [0.18–0.24]	2–
			Moderate: *ρ* = 0.25 [0.22–0.28]	2–
			Vigorous: *ρ* = 0.24 [0.21–0.26]	2–
			Walking: *ρ* = 0.21 [0.18–0.24]	2–
			Cycling: *ρ* = 0.15 [0.12–0.18]	2–
			Sports: *ρ* = 0.22 [0.19–0.25]	2–
			Gardening: *ρ* = 0.16 [0.13–0.19]	2–
			Do-it-yourself activities: *ρ* = 0.15 [0.12–0.18]	2−
			Housework: *ρ* = 0.09 [0.05–0.12]	2−
			Other: *ρ* = 0.07 [0.04–0.10]	2−
			Classification(tertiles): *κ* = 0.16	
MVPA questions Swedish version Ekblom et al. [52]	948	Accelerometer (ActiGraph GT3X and GT3X+, hip, waking hours of 7 days [4 days], 60s, Sasaki et al. [144])	Moderate-to-vigorous: *ρ* = 0.14	1−
			Classification (active/inactive): AUC = 0.57 [0.54–0.63], Sensitivity = 62%, Specificity = 56%	
			Moderate-to-vigorous: Median difference = − 21, 5th to 95th percentile: − 81 to 111 (min/day)	
NC85+PAQ English version Innerd et al. [61]	337	Accelerometer (GENEA, wrist, 7 days [5 days], Esliger et al. [145])	Total (low active group): *ρ* = 0.10	1−
			Total (moderate active group): *ρ* = 0.38	1−
			Total (high active group): *ρ* = 0.34	1−
NPAQ German version Bödeker et al. [47]	58	Pedometer (HJ-720 IT-E2, waking hours of 7 days [3 days])	Total: *ρ* = 0.45 [0.21–0.76]	3+
			Moderate: *ρ* = 0.22 [− 0.10 to 0.56]	3−
			Vigorous: *ρ* = − 0.05 [− 0.40 to 0.50]	3−
			Moderate-to-vigorous: *ρ* = 0.15 [− 0.18 to 0.48]	3−
			Walking (total): *ρ* = 0.49 [0.24–0.82]	1−
			Walking (within neighborhood): *ρ* = 0.43 [0.16–0.76]	1−
			Walking (within neighborhood for transport): *ρ* = 0.20 [− 0.11 to 0.51]	1−
			Walking (within neighborhood for recreation): *ρ* = 0.61 [0.41–0.998]	1−
			Walking (outside neighborhood): *ρ* = 0.29 [− 0.001 to 0.60]	1−
			Walking (outside neighborhood for transport): *ρ* = 0.21 [− 0.10 to 0.53]	1−
			Walking (outside neighborhood for recreation): *ρ* = 0.52 [0.23–0.91]	1−
			Walking for transport (in and outside): *ρ* = 0.25 [− 0.35 to 0.55]	1−
			Walking for recreation (in and outside): *ρ* = 0.44 [0.17–0.77]	1−
			Total biking: *ρ* = 0.33 [0.05–0.64]	3−
PASB-Q English version Fowles et al. [54]	32	Accelerometer (ActiGraph GT3X, hip, waking hours of 7 days [4 days], Freedson et al. [136])	Moderate-to-vigorous (PAVS): *r* = 0.50	1+
			Classification (active/inactive): Sensitivity = 60%, Specificity = 83%	
			Moderate-to-vigorous (PAVS): LOA = − 188 to 288 (min/week)	
PASE Turkish version Ayvat et al. [81]	80	Questionnaire (IPAQ-LF)	Total: *r* = 0.74	3+
			Leisure time activity: *r* = 0.68	3−
			Household activity: *r* = 0.65	3−
			Work-related activity: *r* = 0.57	3−
PAVS English version Ball et al. [45]	269	Questionnaire (Modifiable Activity Questionnaire)	Moderate-to-vigorous: *r* = 0.71	3+
			Classification (active/inactive): *κ* = 0.55 [0.45–0.64]	
			Moderate-to-vigorous: $\bar{d}$ = − 86.3, LOA = − 371 to 199 (min/week)	
PHAS question Swedish version Ekblom et al. [52]	948	Accelerometer (ActiGraph GT3X and GT3X+, hip, waking hours of 7 days [4 days], 60 s, Sasaki et al. [145])	Total (LTPA): *ρ* = 0.26	2−
			Classification (active/inactive): AUC = 0.70 [0.66–0.74], Sensitivity = 92%, Specificity = 27%	
QAPPA French version de Souto Barreto [70]	265	Questionnaire (exercise behavior [yes/no] in the last 2 months)	Moderate (PAEE): Significant difference between exercisers and non-exercisers (Wilcoxon rank sum test)	Not rated
			Vigorous (PAEE): Significant difference between exercisers and non-exercisers (Wilcoxon rank sum test)	Not rated
			Moderate-to-vigorous (PAEE): Significant difference between exercisers and non-exercisers (Wilcoxon rank sum test)	Not rated
			Classification (active/inactive): Significant difference between exercisers and non-exercisers (Chi-squared test)	
SGPALS (LT question) Swedish version Ekblom et al. [52]	948	Accelerometer (ActiGraph GT3X and GT3X+, hip, waking hours of 7 days [4 days], 60 s, Sasaki et al. [144])	Total (LTPA): *ρ* = 0.21	2−
			Classification (active/inactive): AUC = 0.64 [0.59–0.68], Sensitivity = 55%, Specificity = 70%	
Single item on recreational and domestic activity English version Jefferis et al. [62]	1377	Accelerometer (Actigraph, GT3X, hip, waking hours of 7 days [3 days], Copeland et al. [138])	Total (domestic/recreational PA, compared to cpm; steps; MVPA): *ρ* = 0.46; *ρ* = 0.45; *ρ* = 0.43	2+ 3+ 3+
Walking question Swedish version Ekblom et al. [52]	948	Accelerometer (ActiGraph GT3X and GT3X+, hip, waking hours of 7 days [4 days], 60 s, Sasaki et al. [144])	Walking: *ρ* = 0.26	2−
			Classification (active/inactive): AUC = 0.61 [0.55–0.66], Sensitivity = 70%, Specificity = 48%	
WHI-PAQ^g^ English version Neuhouser et al. [65]	450	DLW	Total (PAEE): *R*^2^ = 3.4% (10.7% when corrected for biomarker measurement error)	1−
WHS-AASPA English version Shiroma et al. [68]	10,115	Accelerometer (ActiGraph GT3X+, hip, waking hours of 7 days, Copeland et al. [138], Freedson et al. [136], Matthews et al. [140], Sasaki et al. [144])	Moderate-to-vigorous^h^: *ρ* = 0.35 [0.33–0.37]; *ρ* = 0.36 [0.35–0.38]; *ρ* = 0.39 [0.37–0.40]; *ρ* = 0.37 [0.36–0.39]	1− 1− 1− 1−
			*No minimum bout length*	
			Classification (active/inactive)^h^: *κ* = 0.09; *κ* = 0.21; *κ* = 0.18; *κ* = 0.25	
			*10-min minimum bout length*	
			Classification (active/inactive)^h^: *κ* = 0.25; *κ* = 0.22, *κ* = 0.11; *κ* = 0.15	
ZPAQ English version Harris et al. [57]	234	Accelerometer (Actigraph GT1M, hip, 7 days [5 days], 5 s)	Total (excluding household): *r* = 0.35	2−
ZPAQ Modified English version Harris et al. [57]	234	Accelerometer (Actigraph GT1M, hip, 7 days [5 days], 5 s)	Total (including household): *r* = 0.34	2−
	121	Pedometer (Yamax Digi-walker SW-200, hip, 7 days [5 days])	Total (including household): *r* = 0.36	3−

*AAFQ* Arizona Activity Frequency Questionnaire, *AAS* Active Australia Survey, *ACLS-PALS* Aerobic Center Longitudinal Study—Physical Activity Long Survey, *ACLS-PASS* Aerobic Center Longitudinal Study—Physical Activity Short Survey, *Active-Q* Web-based Physical Activity Questionnaire Active-Q, *AUC* area under the curve, *BRHS* British Regional Heart Study Physical Activity Questionnaire, *CHAMPS* Community Health Activities Model Program for Seniors, *cpm* counts per minute, $\bar{d}$ change in the mean, *DLW* doubly labeled water, *EPAQ2* Norfolk cohort of the European Prospective Investigation into Cancer (EPIC-Norfolk) Physical Activity Questionnaire, EPIC European Prospective Investigation into Cancer, *GPPAQ* General Practice Physical Activity Questionnaire, *h* hours, *IPAQ-E* International Physical Activity Questionnaire for the Elderly, *IPAQ-LF* International Physical Activity Questionnaire—long-form, *IPAQ-SF* International Physical Activity Questionnaire—short-form, *κ* Kappa coefficient, *kcal* kilocalories, *kg* kilogram, *kj* kilojoules, *LAPAQ* Longitudinal Aging Study Amsterdam Physical Activity Questionnaire, *LOA* limits of agreement, *log* logarithm, *LT* leisure time, *LTPA* leisure time physical activity, *min* minutes, *MET* metabolic equivalent, *mLTPA-Q* Modified Leisure Time Physical Activity Questionnaire, *Modified Minnesota LTPA-Q* Modified version of the Minnesota Leisure Time Physical Activity Questionnaire, *MVPA* moderate-to-vigorous physical activity, *NC85+PAQ* Newcastle 85+ Study Physical Activity Questionnaire*, NPAQ* Neighborhood Physical Activity Questionnaire, *PA* physical activity*, PAEE* physical activity energy expenditure, *PAI* physical activity index*, PASB-Q* Physical Activity and Sedentary Behavior Questionnaire, *PASE* Physical Activity Scale for the Elderly, *PAVS* Physical Activity Vital Sign Questionnaire, *PHAS question* Public Health Agency of Sweden physical activity question, *φ* phi correlation coefficient, QAPPA Questionnaire d’Activité Physique pour les Personnes Âgées (Physical Activity Questionnaire for the Elderly), *r* Pearson correlation coefficient, *R*^2^ R-squared, *ρ* Spearman correlation coefficient, *s* seconds, *SGPALS* Saltin-Grimby Physical Activity Level Scale, *WHI-PAQ* Women’s Health Initiative Physical Activity Questionnaire, *WHS-AASPA* Women’s Health Study: Accelerometer Ancillary Study Physical Activity Form, *ZPAQ* Zutphen Physical Activity Questionnaire

^a^As described in Sect. 2.5, the quality of the individual study was evaluated per questionnaire and construct/dimension of PA and can be either very good (1), adequate (2), doubtful (3) or inadequate (4). Additionally, the reported results were rated (i.e., sufficient [+], insufficient [–]) as described in Sect. 2.4

^b^Results based on the first group (home based); second group (group exercise)

^c^Results based on second group (group exercise)

^d^Based on the reported results, we calculated the LOA using the formula LOA = $\bar{d}$ ± 1.96*s*$\surd 2$, where s = within-subject standard deviation (typical error) [146]

^e^The comparison was considered of high quality due to combined sensing and individual calibration

^f^Results based on different lower and upper accelerometer cut points: 760–2019 counts/min; 2020–4944 counts/min; 760–4944 counts/min

^g^Results based on both recreational and household-related PA. However, information about household-related PA was obtained from a previous data collection wave

^h^Results based on different accelerometer cut points: 760 cpm (vertical axis) [140]; 1041 cpm (vertical axis) [138]; 1952 cpm (vertical axis) [136]; 2690 cpm (triaxial) [144]

In at least one study, versions of 13 questionnaires (AAS, ACLS-PALS, ACLS-PASS, BRHS, CHAMPS, IPAQ-LF, mLTPA-Q, Neighborhood Physical Activity Questionnaire (NPAQ), PAQ-EJ, PASB-Q, PASE, PAVS, Single item on Recreational and Domestic Activity) showed sufficient hypotheses testing for construct validity in assessing the overall construct (e.g., total PA, total LTPA) and/or subdimensions (i.e., MVPA, walking) of PA. The results for the SBAS [99] and QAPPA [70] were not rated because the authors reported p-values rather than effect sizes. Similarly, the results for the General Practice Physical Activity Questionnaire (GPPAQ) [44] were not rated since no combined effect size for sensitivity and specificity was reported [e.g., area under the curve (AUC)]. The responsiveness of the AAS for the assessment of MVPA and other subdimensions of PA was insufficient.

### Quality of the Body of Evidence

The quality of the body of evidence (i.e., all studies from the previous review and update combined) together with the rating of measurement properties for all available self-administered questionnaires assessing PA in older adults is shown in Table 6. None of the included questionnaires provided evidence for all relevant measurement properties (reliability, measurement error, hypotheses testing for construct validity, responsiveness). Overall, the quality of evidence for both sufficient and insufficient measurement properties was often low to moderate. The CHAMPS, IPAQ-SF and PASE were the most frequently assessed.

**Table 6:** GRADE evidence profile: measurement properties of all available self-administered PA questionnaires in older adults

Measurement property	Construct/dimension per questionnaire	Results	No. of studies (*n*^*a*^)	GRADE				
				Risk of bias	Inconsistency	Indirectness	Imprecision	Quality of evidence
Reliability								
	Active-Q Swedish version							
	MVPA	−	1 (148) [48]	None	–	Serious^b^	None	Moderate
	Cambridge Index English version							
	Total	−	1 (182) [93]	None	–	None	None	High
	CHAMPS English version^c^							
	Total	−	4 (326) [49, 82, 91, 94]	None	None^d^	None	None	High
	MVPA	+	3 (270) [82, 91, 94]	None	Serious	None	None	Moderate
	CHAMPS Modified English version by Giles et al.							
	MVPA^e^	+	1 (39) [84]	None	–	None	Serious	Moderate
	Walking	+	1 (42) [84]	None	–	None	Serious	Moderate
	CHAMPS Modified English version by Hekler et al.							
	Total	–	1 (748) [59]	Serious	–	None	None	Moderate
	MVPA	–	1 (748) [59]	Serious	–	None	None	Moderate
	EPIC English version							
	Total^f^	−	1 (182) [93]	None	–	None	None	High
	FPACQ Flemish version							
	Total	**+**	1 (36) [87]	None	–	None	Serious	Moderate
	GPPAQ English version							
	Total	−	1 (126) [44]	None	–	None	None	High
	IPAQ-LF Serbian version							
	Total	+	1 (660) [64]	None	–	None	None	High
	Walking	−	1 (660) [64]	None	–	None	None	High
	IPAQ-SF Chinese version							
	Total	+	1 (224) [89]	None	–	None	None	High
	Walking	+	1 (224) [89]	None	–	None	None	High
	IPAQ-SF Japanese version							
	Total	−	1 (325) [72]	None	–	None	None	High
	Walking	−^g^	1 (325) [72]	None	–	None	None	High
	IPEQ English version							
	Total	+	1 (50) [51]	None	–	None	None	High
	LAPAQ Dutch version							
	Total	−	1 (86) [69]	Serious	–	None	None	Moderate
	mLTPA-Q English version							
	MVPA	−	1 (35) [54]	Serious	–	None	Serious	Low
	Modified Baecke Dutch version							
	Total	+^h^	1 (30) [86]	Serious	–	Serious^b^	Serious	Very low
	OA-ESI English version							
	Total	–	2 (46) [95]	Serious	None	None	None	Moderate
	PAQ-EJ Japanese version							
	Total	–	1 (147) [96]	Serious	–	None	None	Moderate
	MVPA	−	1 (147) [96]	Serious	–	None	None	Moderate
	PASB-Q English version							
	MVPA	+	1 (35) [54]	Serious	–	None	Serious	Low
	PASE All versions							
	Total	+	7 (1064) [66, 73, 76, 92, 79, 81, 97]	None	None^d^	None	None	High
	PASE Chinese version							
	Total	+	2 (98) [66, 73]	None	None	None	None	High
	Walking	−	1 (66) [73]	None	–	Serious^i^	None	Moderate
	PASE English version							
	Total	+	1 (254) [92]	Very serious	–	None	None	Low
	PASE Italian version							
	Total	+	1 (48) [79]	None	–	None	None	High
	PASE Japanese version							
	Total	−	1 (257) [97]	Serious	–	None	None	Moderate
	PASE Norwegian version							
	Total	+	1 (327) [76]	None	–	None	None	High
	PASE Persian version							
	Walking	+	1 (278) [80]	None	–	None	None	High
	PASE Turkish version							
	Total	+	1 (80) [81]	None	–	None	None	High
	QAPPA French version							
	MVPA	−	1 (225) [70]	Serious	–	None	None	Moderate
	QAPSE French version							
	MVPA	+	1 (44) [85]	Serious	–	None	Serious	Low
	SBAS English version							
	Total	−	1 (996) [71]	Very serious	–	None	None	Low
	Self-administered PAQ Swedish version							
	Total	−	2 (414) [75, 90]	None	None	None	None	High
	WHI-PAQ English version^i^							
	Total	+	1 (569) [88]	None	–	Serious^b^	None	Moderate
	MVPA	+	1 (569) [88]	None	–	Serious^b^	None	Moderate
	Walking	+	1 (569) [88]	None	–	Serious^b^	None	Moderate
Measurement error								
	CHAMPS English version^c^							
	Total	−	1 (56) [49]	None	–	None	None	High
	EPIC English version							
	Total^k^	−	1 (182) [93]	None	–	None	None	High
	LAPAQ Dutch version							
	Total	−	1 (86) [69]	None	–	None	None	High
	PASE Chinese version							
	Total	−	1 (66) [73]	None	–	Serious^i^	None	Moderate
Hypotheses testing for construct validity								
	AAFQ English version							
	Total	−	1 (450) [65]	None	–	Serious^b^	None	Moderate
	AAS English version							
	Total	+	2 (89) [55, 58]	Serious	None	None	Serious	Low
	MVPA	−	2 (368) [58, 77]	None	None	None	None	High
	Walking	−	1 (50) [58]	None	–	None	Serious	Moderate
	ACLS-PALS English version							
	MVPA	+^l^	1 (71) [46]	None	–	None	Serious	Moderate
	ACLS-PASS English version							
	MVPA	+^l^	1 (71) [46]	None	–	None	Serious	Moderate
	Active-Q Swedish version							
	MVPA	−	1 (148) [48]	None	–	Serious^b^	None	Moderate
	BRHS English version							
	Total	+	1 (1377) [62]	None^m^	–	Serious^b^	None	Moderate
	Cambridge Index English version							
	Total	−	2 (1871) [53, 93]	None	None	None	None	High
	CHAMPS English version^c^							
	Total	−	2 (134) [49, 91]	None	None	None	None	High
	MVPA	−	1 (78) [91]	Serious	–	None	Serious	Low
	CHAMPS Modified English version by Giles et al.							
	MVPA^e^	−	1 (38) [84]	Very serious	–	None	Serious	Very low
	Walking	−	1 (44) [84]	None	–	None	Serious	Moderate
	CHAMPS Modified English version by Hekler et al.							
	Total	−	1 (850) [59]	None	–	None	None	High
	MVPA	−	1 (850) [59]	None	–	None	None	High
	EPAQ2 Modified English version							
	Total	−	1 (1689) [53]	None	–	None	None	High
	MVPA	−	1 (1689) [53]	None	–	None	None	High
	EPIC English version							
	Total^f^	−	1 (182) [93]	None	–	None	None	High
	FPACQ Flemish version							
	Total	−	1 (49) [87]	Serious	–	None	Serious	Low
	IPAQ-E Swedish version							
	Walking	−	1 (54) [60]	Serious	–	None	Serious	Low
	IPAQ-LF English version							
	MVPA	+	1 (226) [78]	None	–	None	None	High
	IPAQ-LF Modified Dutch version							
	Total	−	1 (196) [74]	Very serious	–	None	None	Low
	MVPA	−	1 (196) [74]	None	–	None	None	High
	IPAQ-SF All versions							
	Total	−	4 (949) [50, 56, 72, 89]	Serious	None	None	None	Moderate
	Walking	−	3 (657) [56, 72, 89]	None	None	None	None	High
	IPAQ-SF Chinese version							
	Total	−	1 (224) [89]	Very serious	–	None	None	Low
	Walking	−	1 (224) [89]	None	–	None	None	High
	IPAQ-SF English version							
	Total	−	1 (127) [56]	Very serious	–	None	None	Low
	Walking	−	1 (127) [56]	Very serious	–	None	None	Low
	IPAQ-SF Japanese version							
	Total	−^g^	1 (306) [72]	Serious	–	None	None	Moderate
	Walking	−	1 (306) [72]	None	–	None	None	High
	IPAQ-SF Portuguese version							
	Total	−	1 (292) [50]	Very serious	–	Serious^b^	None	Very low
	LAPAQ Dutch version							
	Total	−	2 (1498) [63, 69]	None	None	None	None	High
	mLTPA-Q English version							
	MVPA	+	1 (32) [54]	Very serious	–	None	Serious	Very low
	Modified Baecke Dutch version							
	Total	−	1 (28) [86]	None	–	Very serious^n^	Very serious	Very low
	Modified Minnesota LTPA-Q English version							
	Total	−	1 (3975) [67]	Serious	–	None	None	Moderate
	Walking	−	1 (3975) [67]	Serious	–	None	None	Moderate
	MVPA questions Swedish version							
	MVPA	−	1 (948) [52]	None	–	None	None	High
	NC85+PAQ English version							
	Total	−	1 (337) [61]	None	–	None	None	High
	NPAQ German version							
	Total	+	1 (58) [47]	Very serious	–	None	Serious	Very low
	MVPA	−	1 (58) [47]	Very serious	–	None	Serious	Very low
	Walking	−	1 (58) [47]	None	–	None	Serious	Moderate
	OA-ESI English version							
	Total	−	1 (327)	Very serious	–	Serious^b^	None	Very low
	PAQ-EJ Japanese version							
	Total	+	1 (147) [96]	Very serious	–	None	None	Low
	MVPA	+	1 (147) [96]	None	–	None	None	High
	PASB-Q English version							
	MVPA	+	1 (32) [54]	None	–	None	Serious	Moderate
	PASE Dutch version							
	Total	−	1 (21) [83]	None	–	None	Very serious	Low
	PASE English version							
	Total	+	1 (78) [91]	None	–	None	Serious	Moderate
	PASE Japanese version							
	Total	−	1 (200) [97]	None	–	None	None	High
	PASE Turkish version							
	Total	+	1 (80) [81]	Very serious	–	None	Serious	Very low
	PAVS English version							
	MVPA	+	1 (269) [45]	Very serious	–	Very serious^o^	None	Very low
	PHAS question Swedish version							
	Total	−	1 (948) [52]	Serious	–	None	None	Moderate
	Self-administered PAQ Swedish version							
	Total	−	2 (227) [75, 98]	Serious	None	None	None	Moderate
	SGPALS (LT question) Swedish version							
	Total	−	1 (948) [52]	Serious	–	None	None	Moderate
	Single item on Recreational and Domestic Activity English version							
	Total	+	1 (1377) [62]	Serious	–	Serious^b^	None	Low
	Walking question Swedish version							
	Walking	−	1 (948) [52]	Serious	–	None	None	Moderate
	WHI-PAQ English version^j^							
	Total	−	1 (450) [65]	None	–	Very serious^p^	None	Low
	WHS-AASPA English version							
	MVPA	−	1 (10115) [68]	None	–	Serious^b^	None	Moderate
	ZPAQ English version							
	Total	−	1 (234) [57]	Serious	–	None	None	Moderate
	ZPAQ Modified English version^q^							
	Total	−	1 (234) [57]	Serious	–	None	None	Moderate
Responsiveness								
	AAS English version							
	MVPA	−	1 (238) [77]	None	–	None	None	High

*AAFQ* Arizona Activity Frequency Questionnaire, *AAS* Active Australia Survey, *ACLS-PALS* Aerobic Center Longitudinal Study—Physical Activity Long Survey, *ACLS-PASS* Aerobic Center Longitudinal Study—Physical Activity Short Survey, *Active-Q* Web-based Physical Activity Questionnaire Active-Q, *BRHS* British Regional Heart Study Physical Activity Questionnaire, *CHAMPS* Community Health Activities Model Program for Seniors, *EPAQ2* Norfolk cohort of the European Prospective Investigation into Cancer (EPIC-Norfolk) Physical Activity Questionnaire, EPIC European Prospective Investigation into Cancer, *FPACQ* Flemish Physical Activity Computerized Questionnaire, *GPPAQ* General Practice Physical Activity Questionnaire, *GRADE* Grading of Recommendation, Assessment, Development and Evaluation, *HEPA* health enhancing physical activity*, IPAQ-E* International Physical Activity Questionnaire for the Elderly, *IPAQ-LF* International Physical Activity Questionnaire—long-form, *IPAQ-SF* International Physical Activity Questionnaire—short-form, *IPEQ* Incidental and Planned Exercise Questionnaire, *LAPAQ* Longitudinal Aging Study Amsterdam Physical Activity Questionnaire, *LT* leisure time, *LTPA* leisure time physical activity, *min* minutes, *mLTPA-Q* Modified Leisure Time Physical Activity Questionnaire, *Modified Minnesota LTPA-Q* Modified version of the Minnesota Leisure Time Physical Activity Questionnaire, *MVPA* moderate-to-vigorous physical activity, *NC85+PAQ* Newcastle 85+ Study Physical Activity Questionnaire, *NPAQ* Neighborhood Physical Activity Questionnaire, *OA-ESI* Older Adult Exercise Status Inventory, *PA* physical activity, *PAQ* Physical Activity Questionnaire, *PAQ-EJ* Physical Activity Questionnaire for Elderly Japanese, *PASB-Q* Physical Activity and Sedentary Behavior Questionnaire, *PASE* Physical Activity Scale for the Elderly, *PAVS* Physical Activity Vital Sign Questionnaire, *PHAS question* Public Health Agency of Sweden physical activity question, QAPPA Questionnaire d’Activité Physique pour les Personnes Âgées (Physical Activity Questionnaire for the Elderly), *QAPSE* Questionnaire d’Activité Physique Saint-Etienne, *SBAS* Stanford Brief Activity Survey, *SGPALS* Saltin-Grimby Physical Activity Level Scale, *WHI-PAQ* Women’s Health Initiative Physical Activity Questionnaire, *WHS-AASPA* Women’s Health Study: Accelerometer Ancillary Study Physical Activity Form, *ZPAQ* Zutphen Physical Activity Questionnaire

Results are shown as sufficient (+) or insufficient (−) measurement properties depending on scores and rating obtained from Tables 4 and 5, as well as from Electronic Supplementary Material Table S3 and Electronic Supplementary Material Table S4. Results are shown for the overall construct of the questionnaire (e.g., total PA, total PAEE, total LTPA), also called ‘total’ score, and for the subdimensions MVPA and walking

^a^Total number of participants across all studies

^b^We considered serious indirectness when only women or men were included in the sample

^c^Including only original versions

^d^We did not consider serious inconsistency since the majority of results were consistent and there was only little variability in effects

^e^Based on the HEPA score

^f^Based on the overall PA index (including occupational PA)

^g^Based on the majority of results. There was only a single positive rating in a subsample (male participants of a specific age group)

^h^Based on the shorter interval between test and retest

^i^We considered serious indirectness since only Chinese participants emigrated to Canada (i.e., living in Vancouver for at least 5 years) were included

^j^Results for reliability were based on recreational PA whereas results for hypotheses testing for validity were based on both recreational and household activities. Consequently, results for the two measurement properties cannot be considered for the same questionnaire version

^k^Results for measurement error were based on the continuous score excluding occupational PA in contrast to the results for reliability and hypotheses testing for construct validity which were based on the overall PA index. Consequently, these results cannot be considered for the same construct/dimension

^l^Results were based on the 1-min bout definition since the ACLS-PALS and ACLS-PASS were not designed to measure MVPA occurring in bouts of ≥ 10 min [46, 147]

^m^Results were based on level 2 and level 3 of quality. However, we did not consider serious risk of bias due to the magnitude of effects and the fact, that the comparison with counts per minute (level 1) was almost acceptable

^n^We considered very serious indirectness since only women were included in the sample and the representativeness of the accelerometer measurement period can be questioned (i.e., one day of measuring)

^o^We considered very serious indirectness because the obtained score of the questionnaire differs from the definition of the dimension MVPA. As mentioned by the authors [45], time spent in either moderate or vigorous PA is obtained. Thus, no overall MVPA score can be calculated. Moreover, the context of the study may not represent the typical administration since the questionnaire was administered during a clinic visit in waiting areas. However, this questionnaire was developed to be a brief measure of PA during regular clinic visits

^p^Very serious indirectness was considered since only women were included in the sample and additional information about the construct (e.g., household/yard PA) was not collected during the study but obtained from a previous data collection wave

^q^This modified version includes household activities in contrast to the original version [57]

In addition to the evidence provided for each questionnaire version, we considered summarizing the results from multiple studies on eight questionnaires (AAS, Cambridge Index, CHAMPS, IPAQ-LF, IPAQ-SF, LAPAQ, OA-ESI, PASE). Regarding reliability and measurement error, results from studies on versions of the IPAQ-SF and PASE (i.e., for the assessment of walking only) were not summarized due to the observed inconsistency in results. Likewise, we did not summarize the results on hypotheses testing for construct validity on versions of the IPAQ-LF and PASE. It is likely that these inconsistent results can be explained by cultural adaptations and modifications of the questionnaire. Results of versions of the ZPAQ were not summarized because they were assessed in the same sample. Two studies [59, 84] assessed modified English versions of the CHAMPS. Because of moderate-to-strong modifications of the original questionnaire (e.g., replacing items; see Sect. 3.1), we considered these versions as different instruments and provided the quality of evidence separately.

Several limitations associated with the quality of evidence were observed. First, for some questionnaires, serious indirectness was considered when the evidence was based on a single study including only women or men (e.g., BRHS) [62]. Second, sometimes, a positive result was only reported in a subsample of participants such as in men at older age [e.g., reliability of the IPAQ-SF (Japanese version) for the assessment of walking [72]]. Furthermore, some studies reported results based on different levels of quality (e.g., very good and doubtful). If this was the case, we considered results based on higher quality for the grading. For example, one study [49] aimed to investigate the agreement between PAEE estimated by the CHAMPS and DLW and also presented results compared to the accelerometer. Although the comparison to the accelerometer was sufficient, we used the results based on DLW for the evaluation of the quality of evidence. The use of modified versions and selective reporting of results across different measurement properties resulted in the disadvantage that the evidence could not be considered for the same questionnaire. For instance, two studies [65, 88] evaluated the measurement properties of the WHI-PAQ. However, the evidence cannot be considered together because the results for hypotheses testing for construct validity were based on both recreational and household-related PA [65], but results for reliability were reported separately for these domains [88]. Finally, the different measurement properties were assessed across a variety of language versions (e.g., reliability of the IPAQ-LF was assessed for the Serbian version but information about hypotheses testing for construct validity was available only for other languages).

Regarding the overall construct, there was at least low-quality evidence that versions of six questionnaires (FPACQ, IPAQ-LF, IPAQ-SF, IPEQ, PASE, WHI-PAQ) showed sufficient reliability and versions of five questionnaires (AAS, BRHS, PAQ-EJ, PASE, Single item on Recreational and Domestic Activity) showed sufficient hypotheses testing for construct validity. Versions of two questionnaires provided also either sufficient reliability (Modified Baecke) or hypotheses testing for construct validity (NPAQ), but this was based on very-low-quality evidence. There was moderate-to-high-quality evidence that the measurement error for the overall construct was insufficient for versions of four questionnaires (CHAMPS, EPIC, LAPAQ, PASE).

Regarding the measurement of MVPA, there was at least low-quality evidence that versions of four questionnaires (CHAMPS, PASB-Q, QAPSE, WHI-PAQ) had sufficient reliability and versions of five questionnaires (ACLS-PALS, ACLS-PASS, IPAQ-LF, PAQ-EJ, PASB-Q) had sufficient hypotheses testing for construct validity. Versions of two questionnaires (mLTPA-Q, PAVS) showed also sufficient hypotheses testing for construct validity, but this was based on very-low-quality evidence. There was high-quality evidence for insufficient responsiveness of the AAS in assessing MVPA.

Regarding the measurement of walking, there was at least low-quality evidence that versions of four questionnaires (CHAMPS, IPAQ-SF, PASE, WHI-PAQ) showed sufficient reliability but there was no evidence for sufficient hypotheses testing for construct validity. Overall, corresponding versions of two questionnaires showed both sufficient reliability and hypotheses testing for construct validity, namely the PASE (i.e., English version, Turkish version) concerning the assessment of total PA, and the PASB-Q (English version) concerning the assessment of MVPA. The quality of evidence for these results ranged from very low to high.

## Discussion

The present review is an update of a previous review published in 2010 [28] and aimed to evaluate the measurement properties of all available self-administered PA questionnaires for older adults and to provide recommendations for the most-qualified questionnaires based on the quality of the body of evidence.

The overall evidence of measurement properties for questionnaires assessing PA in older adults is often of low to moderate quality. None of the included questionnaires provided evidence for all relevant measurement properties (reliability, measurement error, hypotheses testing for construct validity, responsiveness). For versions of 14 questionnaires (Active-Q, Cambridge Index, CHAMPS, EPIC, FPACQ, IPAQ-SF, LAPAQ, mLTPA-Q, Modified Baecke, OA-ESI, PAQ-EJ, PASB-Q, PASE, Self-administered PAQ) combined evidence (i.e., on the same version) for reliability and hypotheses testing for construct validity was available. Of these, there was very-low-to-high-quality evidence of both sufficient reliability and hypotheses testing for construct validity for one questionnaire [PASE (English version, Turkish version)] regarding the measurement of total PA, and for another questionnaire [PASB-Q (English version)] regarding the measurement of MVPA. These two questionnaires also met our criteria for sufficient content validity.

The quality of individual studies was often very good or adequate. Only few studies used inadequate statistical approaches such as Pearson or Spearman correlation coefficients for reliability analyses [36, 102]. Although the ICC is the preferred method [36], a low coefficient does not necessarily indicate low reliability. Correlation coefficients are susceptible to several influences such as the variability of PA behaviors (heterogeneity), differences in the shape of the distribution and non-linearity [103, 104]. For example, any serious lack of variability in the sample (e.g., one may consider PA levels of the very old or other subgroups) could have reduced the observed coefficient. Therefore, we recommend considering the limitations of correlation coefficients when interpreting results concerning both reliability and hypotheses testing for construct validity.

The choice of the comparison measure and use of different intensity levels of PA often reduced the quality of the individual study. For example, both accelerometers and pedometers were often used to test hypotheses for construct validity. Although pedometers can be considered as the reference to measure daily steps, they are unable to capture frequency, duration and intensity of PA [105]. Thus, they can be considered as the best choice to evaluate walking but not MVPA or total PA measured by a questionnaire [e.g., IPAQ-SF (Portuguese version) [50]]. In other studies (e.g., on the Modified Minnesota LTPA-Q [67]), moderate PA measured by the questionnaire was compared to total PA from the accelerometer (including also light and vigorous PA). In this case, the best comparison measure would also be moderate PA due to highest similarity to the construct [106]. The need to choose comparison measures as similar as possible was also demonstrated by studies using novel statistical approaches to handle accelerometer data [107]. Specifically, it was shown that the correlation was much lower for distal (light and vigorous PA), compared to proximal PA intensity levels. However, calculating the time spent in different intensity levels using accelerometer data is clearly challenging because of the dependency on intensity-specific cut points [106].

We observed considerable heterogeneity in the collection, processing and reporting of accelerometer data among individual studies. Although most studies considered a 7-day registration period, a broad range of different cut points, epoch lengths (e.g., 5–60 s) and criteria for a valid week (e.g., 1–14 days) were used. These decision rules will impact the obtained PA estimates [108]. Several studies (e.g., on the AAS [55], mLTPA-Q and PASB-Q [54]) did not use population-specific intensity cut points which may result in an under- or overestimation of time spent in different intensity levels [109]. Another shortcoming was that not all studies reported all decisions such as sampling frequency, non-wear definition and use of filters [110]. Therefore, the use of standards for the design of studies on measurement properties of PA questionnaires (e.g., COSMIN study design checklist) [111, 112] is highly recommended. Likewise, experts in the field emphasized the need for standards for using and reporting accelerometer data [106, 113, 114]. However, despite some attempts [110, 115, 116], it seems that there is currently no consensus on the most appropriate use of accelerometers in older adults [117].

Not only the comparison measure, but also PA questionnaires themselves have important limitations which must be considered. Reporting errors can result from problems in recalling the duration of activities, differences in the interpretation of their intensity [38], social desirability [118] or telescoping of events [119]. Moreover, the accuracy of the recall is influenced by factors such as age, weight status, education and mental health [120, 121]. This is problematic when using questionnaires to define dose–response patterns with health outcomes and strongly reduces the comparability of results among studies with different populations. Hence, it is important to consider advantages and disadvantages of each measurement instrument (e.g., questionnaire, accelerometer, pedometer) when selecting a tool for a particular purpose [11].

Many studies used MET values to estimate the energy costs of activities [i.e., to obtain (rates of) PAEE]. These values are multiples of an adult’s average resting metabolic rate (energy expenditure at rest) [122] and are usually obtained from a compendium of physical activities [123, 124]. However, as emphasized by the authors [124], the compendium does not provide specific energy costs of activities for older adults. So far, there exists no comparable list for older adults although recent studies demonstrated that MET values obtained from daily activities of older adults differed considerably to those listed in the compendium [125], including a strong inter-subject variability and a decrease in resting metabolic rate with age [126]. Therefore, the error associated with the universal application of MET values will likely increase when values from a different population will be applied to older adults [127]. It follows that experts in the field have called for studies of subgroup-specific MET values (e.g., regarding age, sex, body mass, disease status) and questioned the accuracy of conventional MET values to describe the energy costs of activities in older adults [128].

After combining the studies from the previous review and our update, we observed serious shortcomings associated with the quality of the body of evidence. First, only one study assessed the responsiveness of a PA questionnaire. Questionnaires are commonly applied in intervention studies in older adults [12] and sufficient responsiveness is indispensable to accurately measure changes of PA over time [36]. Secondly, only three studies [49, 65, 83] used DLW as a comparison method although (rates of) PAEE was often estimated. Furthermore, for most questionnaire versions, there was only a single study available. This often decreased the overall quality of evidence, especially when this study was of lower quality, the sample size was small or the sample was too restricted (e.g., only women). Finally, we also observed inconsistency in the results when trying to summarize the results from multiple studies on different language versions (e.g., reliability of the Chinese and Japanese version of the IPAQ-SF [72, 89]). The varying results (sufficient, insufficient) of different language versions can partly be explained by cultural adaptations and differences in the conceptualisation and interpretation of PA [129]. If inconsistency in the results is observed and/or studies on the cross-cultural validity revealed important differences between the versions, these language versions should be treated separately. Despite careful cross-cultural adaptation, sufficient measurement quality in one version does not guarantee the same quality for other languages and populations [18, 33].

More than half (i.e., 22 of 40) of all questionnaires met our principal criteria for sufficient content validity. Older adults engage in less exercise-related behaviors; whereas low-to-moderate-intensity activities such as walking and gardening become more prevalent [130]. Nevertheless, these light activities are under-represented in available PA questionnaires for older adults and there is a lack of consensus on the conceptualisation of PA in this population [131, 132]. Light activities are less reliably reported than higher intensity activities which outlines a challenge for the measurement of PA in older adults using self-reports [38]. We recommend that the included questionnaires are further appraised with respect to these considerations, as suggested earlier [131, 132].

Whenever assessed, absolute measurement errors were large (e.g., > 2000 min for total PA of the LAPAQ [69]). Although researchers may define a different MIC, it seems that the ability of questionnaires to detect important changes of PA beyond measurement error is limited [36]. Moreover, we observed a substantial lack of absolute agreement between the questionnaire and the comparison measure (usually the accelerometer), such as for the mLTPA-Q (LOA = − 223 to 262 min per week) [54]. This means that the two instruments do not assess the same absolute dose of PA. However, because of a missing gold standard for the measurement of PA [25, 34], the interpretation of these absolute agreements for construct validity is flawed. We simply do not know what the true dose of PA was. Absolute agreements can only be interpreted when a reference method is available, for instance, when total EE estimated by the questionnaire or accelerometer is compared to the accepted standard of DLW [11].

Of the overall body of evidence, versions of the CHAMPS, IPAQ-SF and PASE were assessed the most often. A great number of results were based on low- or very-low-quality evidence which means that we cannot be confident in the observed measurement properties. Lower quality of the evidence was often related to the reliance on single studies with serious shortcomings in quality, sample size or indirectness. Some results (e.g., for total PA, MVPA) were slightly below [e.g., reliability of the Self-Administered PAQ (Swedish version) [90], hypotheses testing for construct validity of the CHAMPS (English version) [91] and PASE (Dutch version) [83]] or above [e.g., reliability of the IPAQ-LF (Serbian version) [64], hypotheses testing for construct validity of the PAVS [45]] our acceptance levels. These results, if based on high-quality evidence, should not be entirely disregarded when selecting a questionnaire to measure PA in older adults.

### Recommendations for Choosing a Questionnaire

The purpose of the study guides the choice of the questionnaire. In addition to earlier recommendations [36], we suggest the following for the selection of a questionnaire to measure PA in older adults:

- Choose a questionnaire which provides sufficient content validity for a particular purpose and evaluate the content of the questionnaire before using it. For instance, we observed noticeable differences not only in format but also in the obtained information (e.g., frequency, duration or intensity may not be obtained for all included activities). Some attempts regarding the evaluation of content validity have been made previously [131, 132]. If the content validity is insufficient, evaluation of further measurement properties is irrelevant [18].
- When measuring total PA, the questionnaire should include all relevant domains of PA (household, recreation, sports, transport). Occupational PA can be seen as optional in older adults, depending on the target population and type of work (e.g., retired people, voluntary work).
- The questionnaire should include at least parameters of frequency and duration of PA and a representative list of light-to-moderate activities which are more frequently performed by older adults [130].
- The choice of the recall period depends on several factors such as cognitive demands, intended construct (e.g., usual PA, lifetime PA) and the intensity of activities [38]. For example, experts in the field have called for improvements in PA self-reports by reducing the recall period (e.g., multiple 24 h recalls) [38]. However, until high-quality evidence for superior recall periods is available, we recommend that the recall period should capture at least an entire week when using a single administration.
- Due to serious differences in PAEE in older adults and the lack of age-specific energy costs of activities [128], we recommend not using MET values. Instead, raw units such as total time or time spent in different intensity levels can be used.
- It is important to choose a questionnaire with both sufficient reliability and hypotheses testing for construct validity in the target population (e.g., older adults). Unfortunately, this was not often the case in the past [12]. If the questionnaire is used to measure change in PA, sufficient responsiveness is required.
- We recommend considering modified versions of questionnaires as separate instruments, especially when inconsistent results were observed and/or studies on cross-cultural validity showed critical differences [33]. This may also be the case for different language versions when questions are replaced and/or the wording is changed during cultural adaptations. The same questionnaire may not be equally qualified in different settings and populations of older adults.
- If evidence for the measurement properties of a particular modified version is missing, we recommend performing pilot tests.

Not only researchers but also healthcare professionals (e.g., practitioners) are interested in the measurement of PA using questionnaires. In this setting, our recommendations can be followed because they represent general recommendations for the use of questionnaires in order to improve the quality of the measurement. However, further aspects such as clinical feasibility, mode of administration and linkage to electronic record systems should be considered [16]. For instance, clinical feasibility was not part of this review, although included in another review evaluating PA questionnaires in healthcare settings [17]. We propose the following additional recommendations for the use of PA questionnaires in healthcare settings:

- Because the administration should be integrated into the daily workflow, we recommend considering the length of the questionnaire (i.e., time to completion). For this, the PASB-Q may serve as a suitable tool with sufficient measurement properties.
- Healthcare professionals should be aware that the mode of administration likely impacts the obtained results (e.g., interviewer- versus self-administered) [133].
- PA questionnaires show inevitable limitations (e.g., reporting errors due to social desirability or difficulties in recalling the duration of activities) [38, 118] and in this review, only limited high-quality evidence for sufficient measurement properties and usually large measurement errors were observed. Therefore, we recommend bearing in mind that the assessment of PA on the individual level (e.g., determining the PA level of a single patient) is likely associated with large measurement errors.

In general, we recommend using questionnaires with sufficient content validity and at least low-quality evidence for sufficient measurement properties (for at least reliability and hypotheses testing for construct validity) [33]. This was the case for the English versions of the PASE, concerning the assessment of total PA, and PASB-Q, concerning the assessment of MVPA. Also, the Turkish version of the PASE revealed sufficient measurement properties, but the results of hypotheses testing for construct validity were based on very-low-quality evidence. The PASE measures PA over the past 7 days and provides an overall weighted score but does not intend to measure EE [92]. The PASB-Q obtains time spent in MVPA in a typical week [54]. It is a brief measure and does not provide separate information for different domains of PA.

We recommend not using questionnaires with insufficient content validity and/or high-quality evidence for insufficient measurement properties (for at least reliability and hypotheses testing for construct validity) [33]. Hence, we recommend not using the Cambridge Index (English version) for total PA, CHAMPS (English version) for total PA, EPIC (English version) for total PA and the IPAQ-SF (Japanese version) for walking. Several more questionnaires showed insufficient content validity (see Sect. 3.3.1) and would not be recommended. However, future studies performing a comprehensive evaluation of the content validity of these questionnaires are needed in order to be able to give solid recommendations based on only content validity.

### Limitations and Strengths of this Review

We used standardized criteria [36] for the rating of measurement properties which are in accordance with our previous reviews [18, 19, 28–30]. However, the common problem when using cut points like this is dichotomization and loss of information. This can be seen in the results when questionnaires showed results just below or above the cut point. Although one may consider both types of results as acceptable, our cut points represent minimal important criteria for sufficient measurement properties.

The quality of evidence for the measurement properties of many (versions of) questionnaires was limited. Moreover, we observed considerable heterogeneity in the use, analysis and reporting of accelerometer data. We did not use standardized criteria to include these methodological aspects into our quality ratings. Although attempts have been made for certain devices [110], a consensus on the most appropriate use of accelerometers in older adults is lacking [114, 117]. Future reviews may be able to include different decision rules such as epoch length, filter and valid wear time into their assessment. Furthermore, different researchers were involved in the previous review and this update which could have influenced the quality (e.g., level of agreement).

The lack of a gold standard to measure PA resulted in the use of various proxy measures (e.g., accelerometers, pedometers, diaries) to test hypotheses for construct validity. The measurement quality of these instruments varies [25], which means that the construct validity of a PA questionnaire is assessed by comparisons to instruments also showing shortcomings in construct validity. This is a serious problem for any study addressing measurement properties of PA measurement instruments. However, we tried to include differences in the measurement quality of the comparison measure in our quality assessment.

The strengths of this review are that it expands the former evidence [28] and provides the latest recommendations for the use of PA questionnaires in older adults. Data extraction and all assessments were performed independently by at least two researchers. We applied transparent methodological guidelines [33, 36, 43] to assess each result with the same set of criteria as well as to evaluate the quality of individual studies and the overall body of evidence. Finally, we presented all results of the included studies in our tables and, therefore, researchers in the field are invited to discuss the results with regards to their own expertise, probably assigning different criteria.

### Recommendations for Future Research

In 2010 [28], it was recommended that a study should provide a detailed description of the sample and should include at least 50 participants. Such a sample size was considered acceptable to address reliability and hypotheses testing for construct validity [103]. We found that newer studies followed these recommendations. Future studies evaluating the quality of PA questionnaires in older adults should consider the following:

- Because the remaining measurement properties (e.g., reliability, hypotheses testing for construct validity) should only be addressed when the questionnaire has sufficient content validity, we recommend evaluating the content validity of the most promising questionnaires.
- Because many results were based on low-quality evidence and, hence, confidence in these is limited, we recommend evaluating questionnaires for which there is currently only low- or very-low-quality evidence available.
- Because for the majority of questionnaires (> 60%) no combined evidence for reliability and hypotheses testing for construct validity was available, we recommend evaluating questionnaires for which there is currently at least low-quality evidence for sufficiency in one measurement property but information on others is missing.
- We found that many questionnaires were available in only one language (usually English, e.g., PASB-Q). Therefore, we recommend evaluating different language versions of the most promising questionnaires (including correct translation and cultural adaptation).
- Because there was a clear lack of studies assessing responsiveness, we recommend assessing the responsiveness of the most promising questionnaires.
- Because many different (versions of) questionnaires exist, we recommend improving the most promising questionnaires rather than developing new ones [19].
- Because the way we handle accelerometer data influences derived PA estimates [108], we recommend not only working on consensus-based standards but also providing a transparent description of accelerometer data collection and processing rules.
- Due to the observed heterogeneity in the design of studies, we recommend using standards [e.g., COSMIN (http://www.cosmin.nl)] for the study design and evaluation of measurement properties of PA measurement instruments.

## Conclusions

Since our review in 2010 [28], many new PA questionnaires for older adults have been developed. All evidence combined, no questionnaire showed sufficient content validity, reliability, hypotheses testing for construct validity and responsiveness, due to the lack of studies. For most questionnaires, only one study was available, and responsiveness was usually not included in the assessment. The quality of the body of evidence was often reduced. However, two questionnaires (PASB-Q, PASE) can be recommended although the quality of different language versions varied. Because an accepted gold standard to measure PA is missing [34], it is difficult to select the best comparison measure to test hypotheses for construct validity of a questionnaire. We concur with experts in the field that researchers should consider strengths and weaknesses of each instrument, and select the best available comparison measure for a particular construct measured by the questionnaire [11, 134]. For the future, we recommend using existing questionnaires without performing minor modifications to the questionnaire. Rather than developing new questionnaires, we should work on improving existing ones.

## Electronic supplementary material

Below is the link to the electronic supplementary material.

Supplementary material 1 (DOCX 134 kb)

## Data Availability

Data sharing is not applicable to this article as no datasets were generated or analyzed during the current study.
