# Supplementary material for: Current Evidence of Measurement Properties of Physical Activity Questionnaires for Older Adults: An Updated Systematic Review
Source: Sports Med. 2020 Mar 3;50(7):1271–315. doi: 10.1007/s40279-020-01268-x (PMC7305082; doi:10.1007/s40279-020-01268-x)
Supplement: Supplementary file 1 — Supplementary material 1 (DOCX 134 kb) [file 40279_2020_1268_MOESM1_ESM.docx]

Supplementary Materials of

**Article title:** Current Evidence of Measurement Properties of Physical Activity Questionnaires for Older Adults: An Updated Systematic Review

**Journal:** Sports Medicine

**Authors:** Matteo C. Sattler^*^, Johannes Jaunig, Christoph Tösch, Estelle D. Watson, Lidwine B. Mokkink, Pavel Dietz, and Mireille N.M. van Poppel

*Correspondence: matteo.sattler@uni-graz.at

Institute of Sport Science, University of Graz, Graz, Austria

Electronic Supplementary Material Appendix S1

Name: Complete search strategy for PubMed

("motor activity"[Mesh:NoExp] OR Exercise[Mesh] OR Sports[Mesh] OR "Physical Exertion"[Mesh] OR "Early Ambulation"[Mesh] OR "Exercise Therapy"[Mesh] OR Motion[Mesh] OR "Movement Technique"[tiab] OR Motor Activit*[tiab] OR Physical Activit*[tiab] OR Locomotor Activit*[tiab] OR Exercis*[tiab] OR Training[tiab] OR Physical Condition*[tiab] OR “Physical Fitness”[tiab] OR “Physical Endurance”[tiab] OR “Movement Therapy”[tiab] OR “Fitness Training”[tiab] OR “Physical Training”[tiab] OR Plyometric[tiab] OR Weight-Lifting[tiab] OR Weight-Bearing[tiab] OR Running[tiab] OR Jogging[tiab] OR Walk*[tiab] OR Cycle[tiab] OR Cycling[tiab] OR Bicycl*[tiab] OR Rowing[tiab] OR Swim*[tiab] OR Ambulati*[tiab])

AND

(instrumentation[sh] OR methods[sh] OR Validation Studies[pt] OR Comparative Study[pt] OR "psychometrics"[MeSH] OR psychometr*[tiab] OR clinimetr*[tw] OR clinometr*[tw] OR "outcome assessment (health care)"[MeSH] OR outcome assessment[tiab] OR outcome measure*[tw] OR "observer variation"[MeSH] OR observer variation[tiab] OR "Health Status Indicators"[Mesh] OR "reproducibility of results"[MeSH] OR reproducib*[tiab] OR "discriminant analysis"[MeSH] OR reliab*[tiab] OR unreliab*[tiab] OR valid*[tiab] OR coefficient[tiab] OR homogeneity[tiab] OR homogeneous[tiab] OR "internal consistency"[tiab] OR (cronbach*[tiab] AND (alpha[tiab] OR alphas[tiab])) OR (item[tiab] AND (correlation*[tiab] OR selection*[tiab] OR reduction*[tiab])) OR agreement[tiab] OR precision[tiab] OR imprecision[tiab] OR "precise values"[tiab] OR test-retest[tiab] OR (test[tiab] AND retest[tiab]) OR (reliab*[tiab] AND (test[tiab] OR retest[tiab])) OR stability[tiab] OR interrater[tiab] OR inter-rater[tiab] OR intrarater[tiab] OR intra-rater[tiab] OR intertester[tiab] OR inter-tester[tiab] OR intratester[tiab] OR intra-tester[tiab] OR interobserver[tiab] OR inter-observer[tiab] OR intraobserver[tiab] OR intra-observer[tiab] OR intertechnician[tiab] OR inter-technician[tiab] OR intratechnician[tiab] OR intra-technician[tiab] OR interexaminer[tiab] OR inter-examiner[tiab] OR intraexaminer[tiab] OR intra-examiner[tiab] OR interassay[tiab] OR inter-assay[tiab] OR intraassay[tiab] OR intra-assay[tiab] OR interindividual[tiab] OR inter-individual[tiab] OR intraindividual[tiab] OR intra-individual[tiab] OR interparticipant[tiab] OR inter-participant[tiab] OR intraparticipant[tiab] OR intra-participant[tiab] OR kappa[tiab] OR kappa's[tiab] OR kappas[tiab] OR repeatab*[tiab] OR ((replicab*[tiab] OR repeated[tiab]) AND (measure[tiab] OR measures[tiab] OR findings[tiab] OR result[tiab] OR results[tiab] OR test[tiab] OR tests[tiab])) OR generaliza*[tiab] OR generalisa*[tiab] OR concordance[tiab] OR (intraclass[tiab] AND correlation*[tiab]) OR discriminative[tiab] OR "known group"[tiab] OR factor analysis[tiab] OR factor analyses[tiab] OR dimension*[tiab] OR subscale*[tiab] OR (multitrait[tiab] AND scaling[tiab] AND (analysis[tiab] OR analyses[tiab])) OR item discriminant[tiab] OR interscale correlation*[tiab] OR error[tiab] OR errors[tiab] OR "individual variability"[tiab] OR (variability[tiab] AND (analysis[tiab] OR values[tiab])) OR (uncertainty[tiab] AND (measurement[tiab] OR measuring[tiab])) OR "standard error of measurement"[tiab] OR sensitiv*[tiab] OR responsive*[tiab] OR ((minimal[tiab] OR minimally[tiab] OR clinical[tiab] OR clinically[tiab]) AND (important[tiab] OR significant[tiab] OR detectable[tiab]) AND (change[tiab] OR difference[tiab])) OR (small*[tiab] AND (real[tiab] OR detectable[tiab]) AND (change[tiab] OR difference[tiab])) OR meaningful change[tiab] OR "ceiling effect"[tiab] OR "floor effect"[tiab] OR "Item response model"[tiab] OR IRT[tiab] OR Rasch[tiab] OR "Differential item functioning"[tiab] OR DIF[tiab] OR "computer adaptive testing"[tiab] OR "item bank"[tiab] OR "cross-cultural equivalence"[tiab])

AND

((self[tiab] OR child[tiab] OR parent[tiab] OR proxy[tiab]) AND ((report[tiab] OR reported[tiab] OR reporting[tiab]) OR (rated[tiab] OR rating[tiab] OR ratings[tiab]) OR (assessed[tiab] OR assessment[tiab] OR assessments[tiab]))) AND (index[tiab] OR indices[tiab] OR instrument[tiab] OR instruments[tiab] OR measure[tiab] OR measures[tiab] OR questionnaire[tiab] OR questionnaires[tiab] OR profile[tiab] OR profiles[tiab] OR scale[tiab] OR scales[tiab] OR score[tiab] OR scores[tiab] OR status[tiab] OR survey[tiab] OR surveys[tiab])

NOT

(addresses[PT] OR biography[PT] OR “case reports”[PT] OR comment[PT] OR directory[PT] OR editorial[PT] OR festschrift[PT] OR interview[PT] OR lectures[PT] OR “legal cases”[PT] OR legislation[PT] OR letter[PT] OR news[PT] OR “newspaper article”[PT] OR “patient education handout”[PT] OR “popular works”[PT] OR congresses[PT] OR “consensus development conference”[PT] OR “consensus development conference”, nih[PT] OR “practice guideline”[PT]) OR (animals[MeSH Terms] NOT humans[MeSH Terms])

Electronic Supplementary Material Table S1

**Table S1** Explanation of acronyms or abbreviated names of questionnaires, studies on measurement properties and sample characteristics (reassessment of studies included^a^ in the previous review, published in 2010)

| Abbreviation | Full name of questionnaire | Studies on measurement properties | Assessed measurement properties | | | | Comparison measures | Sample  *N* (of consented), n (women), age (years), BMI (kg/m^2^), specific characteristics, nationality |
| --- | --- | --- | --- | --- | --- | --- | --- | --- |
|  |  |  | Reliability | Measurement error | Hypotheses testing for construct validity | Responsiveness |  |  |
| Cambridge index | Simple Physical Activity Index of the European Prospective Investigation into Cancer (EPIC) study | Cust et al. [93]  English version | ● |  | ● |  | Acc | 182 (of 189), 82 ♀, mean age = 57.2, mean BMI = 27.7, 30.4% BMI (18.5-24.9), Australia |
| CHAMPS | Community Health Activities Model Program for Seniors | Cyarto et al.^b^ [94]  English version | ● |  |  |  |  | 43 (of 56), 72.1% ♀, mean age = 77.4 (SD = 6.6), Australia |
|  |  | Harada et al. [91]  English version | ● |  | ● |  | Acc | 87 (N/A), 62% ♀, mean age = 75.0 (SD = 6.0), USA |
|  |  | Stewart et al.^b,c^ [82]  English version | ● |  |  |  |  | 249 (N/A), 63.9% ♀, mean age = 71. (SD = 5.6), intervention trial: *n* = 173, active cohort*: n* = 76, USA |
|  |  | Giles et al. [84]  Modified English version | ● |  | ● |  | Ped | 73 (of 73), 45 ♀, 46.6% age (65-69), 24.7% age (70-74), 28.8% age (≥ 75), Australia |
| EPIC | Questionnaire used in the European Prospective Investigation into Cancer and Nutrition | Cust et al. [93]  English version | ● | ● | ● |  | Acc, Q | 182 (of 189), 82 ♀, mean age = 57.2, mean BMI = 27.7, 30.4% BMI (18.5-24.9), Australia |
| FPACQ | Flemish Physical Activity Computerized Questionnaire (based on the IPAQ-LF and others) | Matton et al. [87]  Flemish version | ● |  | ● |  | Acc+record | 49 (of 49), 19 ♀, mean age ♀ = 65.4 (SD = 5.0), mean age ♂ = 64.5 (SD = 5.5), Belgium |
| IPAQ-SF | International Physical Activity Questionnaire - short-form | Deng et al. [89]  Chinese version | ● |  | ● |  | Ped | 224 (of 230), 148 ♀, mean age ♀ = 63.8 (SD = 5.4), mean age ♂ = 67.8 (SD = 5.4), mean BMI ♀ = 24.0 (SD = 3.3), mean BMI ♂ = 23.9 (SD = 3.2), China |
| Modified Baecke | Modified version of the Baecke Questionnaire | Pols et al. [86]  Dutch version | ● |  | ● |  | Acc, diary | 33 (of 35), all ♀, mean age = 61.2 (SD = 6.7), mean BMI = 25.0 (SD = 3.3), the Netherlands |
| OA-ESI | Older Adult Exercise Status Inventory | O’Brien-Cousins [95]  English version | ● |  | ● |  | Q | Reliability study 1: 17 (N/A), all ♀, mean age = 67, Canada  Reliability study 2: 29 (N/A), both men and women, mean age = 71, Canada  Validity study: 327 (N/A), all ♀, mean age = 77, Canada |
| PAQ-EJ | Physical Activity Questionnaire for Elderly Japanese | Yasunaga et al. [96]  Japanese version | ● |  | ● |  | Acc | 147 (of 4447), 86 ♀, mean age ♀ = 72.8 (SD = 4.6), mean age ♂ = 72.8 (SD = 3.9), mean BMI ♀ = 23.2 (SD = 3.1), mean BMI ♂ = 22.9 (SD = 2.8), Japan |
| PASE | Physical Activity Scale for the Elderly | Schuit et al. [83]  Dutch version |  |  | ● |  | DLW | 21 (of 25), 11 ♀, mean age ♀ = 69.2 (SD = 4.8), mean age ♂ = 70.6 (SD = 3.8), mean BMI ♀ = 24.3 (SD = 3.0), mean BMI ♂ = 26.3 (SD = 2.2), the Netherlands |
|  |  | Harada et al. [91]  English version |  |  | ● |  | Acc | 87 (N/A), 62% ♀, mean age = 75.0 (SD = 6.0), USA |
|  |  | Washburn et al.^b^ [92]  English version | ● |  |  |  |  | 277 (of 227), 57% ♀, mean age = 73.0, USA |
|  |  | Hagiwara et al. [97]  Japanese version | ● |  | ● |  | Acc, Q | 325 (N/A), 191 ♀, mean age = 72.6 (SD = 4.9), Japan |
|  |  | Loland^d^ [76]  Norwegian version | ● |  |  |  |  | 343 (of 500), 185 ♀, mean age = 74.8 (SD = 6.6), Norway |
| Pre-EPIC | Questionnaire preceding EPIC | Pols et al. [86]  Dutch version | ● |  | ● |  | Acc, diary | 33 (of 35), all ♀, mean age = 61.2 (SD = 6.7), mean BMI = 25.0 (SD = 3.3), the Netherlands |
| QAPSE | Questionnaire d`Activité Physique Saint-Etienne | Bonnefoy et al. [85]  French version | ● |  |  |  |  | 65 (of 70), 34 ♀, mean age ♀ = 70.5 (SD = 3.5), mean age ♂ = 71.1 (SD = 4.0), mean percentage body fat ♀ = 34.3 (SD = 3.6), mean percentage body fat ♂ = 25.5 (SD = 4.9), France |
| SBAS | Stanford Brief Activity Survey | Taylor-Piliae et al. [99]  English version |  |  | ● |  | Interview | 1010 (of 1023), 379 ♀, mean age = 65.8 (SD= 2.8), mean BMI = 28.4 (SD = 5.2), USA |
| Self-administered PAQ | Self-administered Physical Activity Questionnaire | Orsini et al. [90]  Swedish version | ● |  |  |  |  | 303 (of 360), all ♀, mean age = 65.2 (SD = 5.6), mean BMI = 25.2 (SD = 3.6), 51% overweight/obese, Sweden |
|  |  | Orsini et al. [98]  Swedish version |  |  | ● |  | Acc, diary | 116 (of 151), all ♀, mean age = 64.4 (SD = 5.6), mean BMI = 26.1 (SD = 4.0), 58% overweight/obese, Sweden |
|  |  | Norman et al.^d^ [75]  Swedish version | ● |  | ● |  | Diary | 224 (N/A), all ♂, mean age = 63.2 (SD = 9.3), mean BMI = 25.8 (SD = 3.2), Sweden |
| WHI-PAQ | Women`s Health Initiative Physical Activity Questionnaire | Meyer et al. [88]  English version | ● |  |  |  |  | 1092 (N/A), all ♀, mean age = 64, 39.7% BMI (normal), 55.0% retired, USA |

*Acc* accelerometer, *BMI* body mass index, *DLW* doubly labeled water, *EPIC* European Prospective Investigation into Cancer, *IPAQ-LF* International Physical Activity, Questionnaire—long form, *N/A* not applicable, *PA* physical activity, *Ped* pedometer, *Q* questionnaire, *SD* standard deviation, *USA* United States of America

^a^Two studies (i.e., Norman et al. [75]; Loland [76]) were not included in the previous review, although published prior to September 2008. The results of the two studies are shown together with previously included studies to allow comparisons

^b^Results for hypotheses testing for construct validity were not included since comparisons were performed with non-PA measures

^c^Results for responsiveness were not included since the change in PA measured by CHAMPS was not compared with a change score measured by other PA measurement instruments. Even though an intervention design was used, no a-priori defined hypothesis about a change score (i.e., expected effect size) was evaluated and we were not able to define a specific hypothesis about important group differences between control group and intervention group

^d^This study was not included in the previous review but found during the reference check of the update

Electronic Supplementary Material Table S2

**Table S2** Description of all included PA questionnaires

| Questionnaire | Target population | Construct | | | | Format | | |
| --- | --- | --- | --- | --- | --- | --- | --- | --- |
|  |  | Dimension | Domains^a^ | Recall period | No. of questions | Parameters^b^ | Scores^c^ | Unit of measurement |
| AAFQ [148] | Adults | TEE, PAEE | Recreation/sports/leisure,  occupational, personal care, home, transport | Last 4 weeks | 68 | F, D, I | Total | MET-h/day,  kJ/day |
| AAS [149] | Adults | LTPA | Recreation/sports/leisure, transport | Last week | 8 | F, D, I | Total, moderate, vigorous, walking | min/week,  sessions/week |
| ACLS-PALS [147] | Adults | Exercise habits and participation | Recreation/sports/leisure | Last 3 months | 6 | F, D, I | Total, activity index (run-walk-jog index) | MET-h/week, activity index |
| ACLS-PASS [147] | Adults | PA participation | All | Last 7 days | 2 | D, I | moderate, vigorous, moderate-to-vigorous | MET-h/week |
| Active-Q [150] | Adults | Habitual PA | Recreation/sports/leisure, occupational, transport (to and from work), household | Last year | 9 - 47 (depending on answers) | F, D | Total, light, sedentary-to-light, moderate, vigorous, moderate-to-vigorous | MET-h/day,  kJ/day |
| BRHS [151] | Male adults | Usual PA | Recreation/sports/leisure, occupational^d^, transport, | Usual activity (unspecific, per week, per month) | 4 | F, D | Total | Total score, activity index (6 categories) |
| Cambridge index [152] | Adults (mid-to-late life) | PA (to rank participants according to EE) | Recreation/sports/leisure, occupational | Last 12 months | 4 | D | Total | Activity index (4 categories) |
| CHAMPS [82] | Older adults (65-90 years) | PA, PAEE (changes in PA/PAEE) | Recreation/sports/leisure, household, transport | Last 4 weeks | 41 | F, D, I | Total, moderate-to-vigorous | MET-h/week, frequency/week |
| EPAQ2 [153] | Adults (mid-to-late life) | PA, PAEE | Recreation/sports/leisure,  occupational, household, transport | Last 12 months | 85 | F, D, I | Total, light, moderate, vigorous, moderate-to-vigorous | MET-h/week |
| GPPAQ [44] | Adults (within primary care) | PA | Sports/exercise, occupational, household^d^, transport^d^ | Last week, unspecific | 7 | D | Total | Activity index (4 categories) |
| IPAQ-E [60] | Older adults | PA | All | Last 7 days | 7 | F, D, I | Total, moderate, vigorous, walking | min/day |
| IPAQ-LF [154] | Adults | PA | Recreation/sports/leisure, occupational, household, transport | Last 7 days, usual week | 31 (27 in latest version [155]) | F, D, I | Total, moderate, vigorous, walking,  leisure, household, occupational, transport | MET-min/week |
| IPAQ-SF [154] | Adults | PA | All | Last 7 days, usual week | 7 | F, D, I | Total, moderate, vigorous, walking | MET-min/week |
| IPEQ [51] | Older adults | PA (planned and incidental) | Sports/exercise, household, transport | Last 3 months, last week | 10 | F, D | Total, incidental PA, walking, planned walking, planned PA, planned exercise | h/week |
| LAPAQ [69, 156] | Older adults | PA | Sports/exercise, household, transport | Last 2 weeks | 18 | F, D, I | Total, mild, moderate, vigorous | min/2 weeks |
| mLTPA-Q [54, 157] | Adults | LTPA | Recreation/sports/leisure | Last 4 weeks | 3 | F, D, I | Total, mild, moderate, strenuous, moderate-to-strenuous | min/week |
| Modified Minnesota LTPA-Q [67, 158] | Adults and older adults | PA (at work and in free time) | Recreation/sports/leisure, occupational, household, transport | Last 4 weeks | 20 | F, D | Total, mild, moderate, vigorous | MET-h/week |
| MVPA questions [52] | N/A | MVPA | All (not specified) | Last 7 days | 6 | F, D, I | moderate-to-vigorous | min/week |
| NC85+PAQ [61] | Older adults (≥ 85 years) | PA | All (not specified) | Unspecific (per week/month) | 3 | F, I | Total | Total score, activity index (3 categories) |
| NPAQ [159] | Adults | PA, walking | Recreation/sports/leisure, transport (within and outside of neighborhood) | Usual week | 35 | F, D, I | Total (overall PA index), walking (for transport, for recreation), cycling, other LT activities | MET-min/week, min/week |
| PASB-Q [161] | Adults | PA (to identify PAVS) | All (not specified) | Typical week | 4 | F, D, I^e^ | moderate-to-vigorous, muscle-strengthening | min/week, frequency/week |
| PASE [92] | Older adults | PA | Recreation/sports/leisure, occupational, household, transport | Last 7 days | 12 | F, D, I | Total | Weighted total score, h/day |
| PAVS [45] | Adults (within primary care) | PA | All (not specified) | Typical week | 2 | F, D, I^f^ | Total, moderate-to-vigorous^f^ | min/week |
| PHAS question [162] | Adults (≥ 16 years) | LTPA | Recreation/sports/leisure | Last 12 months | 1 | - | Total | Activity index (4 categories) |
| QAPPA [163] | Older adults | PA | Recreation/sports/leisure, occupational, household, transport | Last 7 days | 4 | F, D, I | moderate, vigorous, moderate-to-vigorous | MET-min/week |
| SBAS [99] | Older adults | Habitual PA | Recreation/sports/leisure, occupational, household, transport | Last year | 2 | - | Total, moderate-to-vigorous (based on categories) | Activity index (25 categories) |
| SGPALS (LT question)^g^ [52, 164] | Adults | Lifetime LTPA | Recreation/sports/leisure | Last 5 years | 1 | - | Total | Activity index (4 categories) |
| Single item on recreational and domestic activity [62, 151] | Male adults | Recreational and domestic PA | Recreation, household | Unspecific (usually) | 1 | - | Total | Activity index (5 categories) |
| Walking question [52] | N/A | Walking | All (not specified) | Last 7 days | 3 | F, D | Walking | min/week |
| WHI-PAQ^h^ [165] | Postmenopausal women | Usual PA | Recreation/sports/leisure (first form), household (second form) | Unspecific (usually, per week/month/year) | 9 | F, D, I | Total, mild, moderate, strenuous, walking | MET-h/week |
| WHS-AASPA [68, 166] | Female adults | Recreational PA | Recreation/sports/leisure, transport | Unspecific (usually), specific week^i^ | 12 | D | Total, moderate-to-vigorous | MET-h/week, min/week |
| ZPAQ^j^ [57, 101] | Retired men | PA | Recreation/sports/leisure, household^j^, transport | Last week, last month, unspecific | 15 | F, D | Total, light, moderate, heavy | kcal/kg/day,  min/week |

*AAFQ* Arizona Activity Frequency Questionnaire, *AAS* Active Australia Survey, *ACLS-PALS* Aerobic Center Longitudinal Study – Physical Activity Long Survey, *ACLS-PASS* Aerobic Center Longitudinal Study – Physical Activity Short Survey, *Active-Q* Web-based Physical Activity Questionnaire Active-Q, *BRHS* British Regional Heart Study Physical Activity Questionnaire, *CHAMPS* Community Health Activities Model Program for Seniors, *D* duration*, EE* energy expenditure*, EPAQ2* Norfolk cohort of the European Prospective Investigation into Cancer (EPIC-Norfolk) Physical Activity Questionnaire, EPIC European Prospective Investigation into Cancer, *F* frequency, *GPPAQ* General Practice Physical Activity Questionnaire, *h* hour, *I* intensity, *IPAQ-E* International Physical Activity Questionnaire for the Elderly, *IPAQ-LF* International Physical Activity Questionnaire – long-form, *IPAQ-SF* International Physical Activity Questionnaire – short-form, *IPEQ* Incidental and Planned Exercise Questionnaire, *kcal* kilocalories, *kg* kilogram, *kJ* kilojoules, *LAPAQ* Longitudinal Ageing Study Amsterdam Physical Activity Questionnaire, *LT* leisure time, *LTPA* leisure time physical activity*, MET* metabolic equivalent*, min* minutes, *mLTPA-Q* Modified Leisure Time Physical Activity Questionnaire, *Modified Minnesota LTPA-Q* Modified version of the Minnesota Leisure Time Physical Activity Questionnaire, *MVPA* moderate-to-vigorous physical activity, *N/A* not applicable, *NC85+PAQ* Newcastle 85+ Study Physical Activity Questionnaire, *NPAQ* Neighborhood Physical Activity Questionnaire, *PA* physical activity, *PAEE* physical activity energy expenditure*, PASB-Q* Physical Activity and Sedentary Behaviour Questionnaire, *PASE* Physical Activity Scale for the Elderly, *PAVS* Physical Activity Vital Sign, *PHAS question* Public Health Agency of Sweden physical activity question, QAPPA Questionnaire d´Activité Physique pour les Personnes Âgées (Physical Activity Questionnaire for the Elderly), *SBAS* Stanford Brief Activity Survey, *SGPALS* Saltin-Grimby Physical Activity Level Scale, *TEE* total energy expenditure, *WHI-PAQ* Women´s Health Initiative Physical Activity Questionnaire, *WHS-AASPA* Women`s Health Study: Accelerometer Ancillary Study Physical Activity Form, *ZPAQ* Zutphen Physical Activity Questionnaire

^a^The domain transport was included if at least walking was measured

^b^Parameters may not be measured for all questions and/or activities listed in the questionnaire. We recommend inspecting the content of the questionnaire in detail before using it

^c^The listed scores were suggested to be calculated based on studies on the development of the questionnaire. Total refers to the overall score of the questionnaire. Depending on the purpose, this can be either total PA, total PAEE or any other dimension (e.g., LTPA)

^d^Not used for scoring in the original version

^e^Moderate and vigorous PA are obtained using the same question

^f^Information about either light, moderate or vigorous PA is obtained

^g^In the original version of the SGPALS [164], there exists another section asking about occupational activities but this section was not included in the study of Ekblom et al. [52]

^h^Information about household-related and recreational PA is usually obtained by two different forms [88]. In the study of Neuhouser et al. [65], only recreational PA was assessed but information about other activities (including household-related PA) were added from a previous data collection wave

^i^The week when the accelerometer was worn

^j^The original version does not include household-related PA. However, this domain was added to the modified version used in the study of Harris et al. [57]

Electronic Supplementary Material Table S3

**Table S3** Reliability and measurement error of PA questionnaires for older adults (reassessment of studies included^a^ in the previous review, published in 2010)

| Questionnaire | Study population (*n*) for analysis | Interval | Results | Study quality and result rating^b^ | Difference to the assessment in 2010 |
| --- | --- | --- | --- | --- | --- |
| Cambridge index  English version  Cust et al. [93] | 182 | 10 months | Total: κ = 0.66 [0.58–0.74] | 1– | results were not included |
| CHAMPS  English version  Cyarto et al. [94] | 43 | 1 week | Total (PAEE): ICC = 0.75 [0.58–0.86]  Light (PAEE): ICC = 0.66 [0.46–0.80]  Moderate (PAEE): ICC = 0.88 [0.79–0.93]  Vigorous (PAEE): ICC = 0.44 [0.17–0.65]  Moderate-to-vigorous (PAEE): ICC = 0.76 [0.61–0.87]  Classification (active/inactive): κ = 0.68 | 1+  1–  1+  1–  1+ | No rating was given due to the small sample size |
| CHAMPS  English version  Harada et al. [91] | 80 | 2 weeks | Total (PAEE): ICC = 0.62  Moderate-to-vigorous (PAEE): ICC = 0.76 | 1–  1+ | results were not included |
| CHAMPS  English version  Stewart et al. [82] | 147 | 6 months | Total (PAEE): ICC = 0.66  Total (frequency): ICC = 0.62  Moderate-to-vigorous (PAEE): ICC = 0.67  Moderate-to-vigorous (frequency): ICC = 0.58 | 2–  2–  2–  2– | None |
| CHAMPS  Modified English version  Giles et al. [84] | 29 to 46 (n_HEPA(PAEE)_ = 39; n_walking(PAEE)_ = 42 | 1 to 2 weeks | HEPA (frequency): ICC = 0.89 [0.77–0.95]  HEPA (duration): ICC = 0.81 [0.63–0.90]  HEPA (PAEE): ICC = 0.84 [0.69–0.91]  Moderate (frequency): ICC = 0.83 [0.66–0.92]  Moderate (duration): ICC = 0.79 [0.61–0.89]  Moderate (PAEE): ICC = 0.80 [0.63–0.89]  Vigorous (frequency): ICC = 0.86 [0.74–0.93]  Vigorous (duration): ICC = 0.79 [0.61–0.88]  Vigorous (PAEE): ICC = 0.78 [0.59–0.88]  Walking (frequency): ICC = 0.93 [0.86–0.97]  Walking (duration): ICC = 0.83 [0.68–0.91]  Walking (PAEE): ICC = 0.85 [0.71–0.92]  Classification (active/inactive): κ = 0.55 | 1+  1+  1+  1+  1+  1+  1+  1+  1+  1+  1+  1+ | No rating was given due to the small sample size |
| EPIC  English version  Cust et al. [93] | 182 | 10 months | Total (including occupational; index): κ = 0.62 [0.53–0.71]  Total (excluding occupational; PAEE): ρ = 0.65 [0.55–0.72]  Light-to-moderate (excluding occupational): ρ = 0.67 [0.58–0.74]  Vigorous (excluding occupational): ρ = 0.71 [0.63–0.78]  Recreational (PAEE): ρ = 0.58 [0.48–0.67]  Household (PAEE): ρ = 0.73 [0.66–0.79]  Measurement error:  Total (excluding occupational; PAEE): *d̄* = – 0.6, LOA^c^ = – 0.6 ± 1.96*59.0 (MET-h/week)  Light-to-moderate (excluding occupational): *d̄* = – 2.2, LOA^c^ = – 2.2 ± 1.96*51.0 (h/week)  Vigorous (excluding occupational): *d̄* = 1.5, LOA^c^ = 1.5 ± 1.96*32.4 (h/week)  Recreational (PAEE): *d̄* = 0.7, LOA^c^ = 0.7 ± 1.96*47.7 (MET-h/week)  Household (PAEE): *d̄* = – 1.3, LOA^c^ = – 1.3 ± 1.96*33.5 (MET-h/week) | 1–  2–  2–  2–  2–  2–  1–  1–  1–  1–  1– | Results for LOA, vigorous, light-to-moderate, recreational and household-realted PA were not included |
| FPACQ  Flemish version  Matton et al. [87] | 36 | 2 weeks | *Women*  Total (duration [Tactl]): ICC = 0.64 [0.24–0.85]  Total (PAL): ICC = 0.77 [047–0.91]  TEE: ICC = 0.96 [0.90–0.99]  Sports (duration): ICC = 0.92 [0.79–0.97]  Transport (duration): ICC = 0.57 [0.14–0.83]  *Men*  Total (duration [Tactl]): ICC = 0.83 [0.62–0.93]  Total (PAL): ICC = 0.89 [0.76–0.96]  TEE: ICC = 0.90 [0.76–0.96]  Sports (duration): ICC = 0.68 [0.37–0.86]  Transport (duration): ICC = 0.81 [0.58–0.92] | 1–  1+  Not assigned  1+  1–  1+  1+  Not assigned  1–  1+ | Levels of quality for TEE were assigned; results for total PA (duration), sports, and transport were not included |
| IPAQ-SF  Chinese version  Deng et al. [89] | 224 | 8 days | Total (PAEE): ICC = 0.84 [0.80–0.87]  Moderate (PAEE): ICC = 0.81 [0.76–0.85]  Vigorous (PAEE): ICC = 0.83 [0.78–0.87]  Walking: ICC = 0.85 [0.81–0.88]  Sitting: ICC = 0.89 [0.86–0.91] | 1+  1+  1+  1+ | None |
| Modified Baecke  Dutch version  Pols et al. [86] | *n*_5 months_ = 30, *n*_11 months_ = 28 | 5 months  11 months | Total: *r* = 0.82  Total: *r* = 0.73 | 2+  2– | No rating was given due to the small sample size |
| OA-ESI  English version  O’Brien-Cousins [95] | 17  29 | ≤ 4 weeks  1 week | Total (Exercise): *r* = 0.34  Moderate (Exercise): *r* = 0.76  Vigorous (Exercise): *r* = 0.51  Total: *r* = 0.77 | 3–  3–  3–  2– | No rating was given due to the small sample size |
| PAQ-EJ  Japanese version  Yasunaga et al. [96] | 147 | 1 month | Total (PAEE): *r* = 0.70  Light (PAEE): *r* = 0.64  Moderate-to-vigorous (PAEE): *r* = 0.71 | 2–  2–  2– | None |
| PASE  English version  Washburn et al. [92] | 254 | 3 to 7 weeks | Total: *r* = 0.84 | 3+ | None |
| PASE  Japanese version  Hagiwara et al. [97] | 257 | 3 to 4 weeks | Total: ICC = 0.65 [0.58–0.72] | 2– | different levels of quality were given |
| PASE  Loland [76]  Norwegian version | 327 | 3 days  3 weeks | Total: *r* = 0.99  Total: *r* = 0.93 | 1+  2+ | This study was not included in the previous review but found during the reference check of the update |
| Pre-EPIC  Dutch version  Pols et al. [86] | *n*_5 months_ = 29, *n*_11 months_ = 28 | 5 months  11 months | TEE: *r* = 0.42  TEE: *r* = 0.60 | Not assigned  Not assigned | Levels of quality for TEE were assigned |
| QAPSE  French version  Bonnefoy et al. [85] | 44 | At least 6 weeks | TEE: *r* = 0.97  Moderate-to-vigorous (PAEE): *r* = 0.86  Professional activities (PAEE): *r* = 0.66  Leisure activities (PAEE): *r* = 0.77  Sports activities (PAEE): *r* = 0.85  Housework (PAEE): *r* = 0.87  Basic daily activities (PAEE): *r* = 0.74  Moving index activities (PAEE): *r* = 0.65 | Not assigned  2+  2–  2–  2+  2+  2–  2– | No rating was given due to the small sample size; different levels of quality were given; levels of quality for TEE were assigned |
| Self-administered PAQ  Swedish version  Orsini et al. [90] | 303 | 1 year | *Obtained score from questionnaire: past year PA*  Total (PAEE): ICC = 0.69 [0.62–0.75]  Walking/cycling (PAEE): ICC = 0.56 [0.48–0.64]  Housework (PAEE): ICC = 0.58 [0.50–0.65]  Occupation (PAEE): ICC = 0.59 [0.51–0.66]  Exercise (PAEE): ICC = 0.49 [0.40–0.58]  Watching TV/reading (PAEE): ICC = 0.59 [0.51–0.67]  Classification (top quintile): Sensitivity = 61%, Specificity = 89%  Classification (bottom quintile): Sensitivity = 54%, Specificity = 88%  Classification of walking/cycling (active/passive): Sensitivity = 69%, Specificity = 78%  Classification of exercise (active/passive): Sensitivity = 65%, Specificity = 86%  Classification of home/household (active/passive): Sensitivity = 75%, Specificity = 85%  Classification of TV/reading (high/low): Sensitivity = 79%, Specificity = 76% | 1–  1–  1–  1–  1– | None |
| Self-administered PAQ  Swedish version  Norman et al. [75] | *n* = 111 | 7 to 12 months | Total (crude score; PAEE): Concordance = 0.66  Total (corrected score; PAEE): Concordance = 0.67  Occupation (PAEE): Concordance = 0.70  Housework (PAEE): Concordance = 0.66  Walking/bicycling/exercise (PAEE): Concordance = 0.61  Watching TV/reading (PAEE): Concordance = 0.67 | 1–  1–  1+  1–  1– | This study was not included in the previous review but found during the reference check of the update |
| WHI-PAQ  English version  Meyer et al. [88] | *n_form 34_* = 569, *n*_form 42_ = 523) | 12 weeks (range: 8–15) | *Form 34 (about recreational/exercise)*  Total (recreational PAEE): ICC = 0.76 [0.71–0.79]  Mild (recreational PAEE): ICC = 0.51 [0.45–0.57]  Moderate (recreational PAEE): ICC = 0.57 [0.52–0.63]  Moderate-to-strenuous (recreational PAEE): ICC = 0.77 [0.73–0.80]  Strenuous (recreational PAEE): ICC = 0.76 [0.73–0.80]  Walking (recreational PAEE): ICC = 0.75 [0.72–0.79]  *Form 42 (about household/yard and sedentary activities)*:  Household (PAEE): ICC = 0.60 [0.55–0.66]  Yard (PAEE): ICC = 0.71 [0.66–0.75]  Sitting/lying (PAEE): ICC = 0.60 [0.54–0.65] | 1+  1–  1–  1+  1+  1+  1–  1+ | None |

*CHAMPS* Community Health Activities Model Program for Seniors, *d̄* change in the mean, EPIC European Prospective Investigation into Cancer, *FPACQ* Flemish Physical Activity Computerized Questionnaire, *h* hour, *HEPA* health enhancing physical activity*, ICC* intraclass correlation coefficient, *IPAQ-SF* International Physical Activity Questionnaire – short-form, *κ* Kappa coefficient, *LOA* limits of agreement, *MET* metabolic equivavlent, *OA-ESI* Older Adult Exercise Status Inventory, *PA* physical activity*, PAL* physical activity level, *PAEE* physical activity energy expenditure, *PAQ* Physical Activity Questionnaire, *PAQ-EJ* Physical Activity Questionnaire for Elderly Japanese, *PASE* Physical Activity Scale for the Elderly, *Pre-Epic* Questionnaire preceding EPIC, *QAPSE* Questionnaire d`Activité Physique Saint-Etienne, *r* Pearson correlation coefficient, *ρ* Spearman correlation coefficient*, TEE* total energy expenditure, *WHI-PAQ* Women´s Health Initiative Physical Activity Questionnaire

^a^Two studies (i.e., Norman et al. [75]; Loland [76]) were not included in the previous review, although published prior to September 2008. The results of the two studies are shown together with previously included studies to allow comparisons

^b^As described in Sect. 2.5, the quality of the individual study was evaluated per questionnaire and construct/dimension of PA and can be either very good (1), adequate (2), doubtful (3) or inadequate (4). Additionally, the reported results were rated (i.e., sufficient [+], insufficient [–]) as described in Sect. 2.4

^c^Based on the reported results, we calculated the LOA using the formula LOA = *d̄* ±1.96*s*$\surd2$, where s = within-subject standard deviation (typical error) [146]

Electronic Supplementary Material Table S4

**Table S4** Hypotheses testing for construct validity and responsiveness of PA questionnaires for older adults (reassessment of studies included^a^ in the previous review, published in 2010)

| Questionnaire | Study population (*n*) for analysis | Comparison measure (type, placement, registration period [valid week], epoch length, cut points) | Results | Study quality and result rating^b^ | Difference to the assessment in 2010 |
| --- | --- | --- | --- | --- | --- |
| Cambridge index  English version  Cust et al. [93] | 182 | Accelerometer (Actigraph model 7164, hip, three times for waking hours of 7 days [4 days], 60 s, Swartz et al. [141]) | Total: ρ = 0.32 [0.19–0.45] | 1– | Results were not included |
| CHAMPS  English version  Harada et al. [91] | n_ankle_ = 56, n_waist_ = 78 | Accelerometer (Mini-Logger Series 2000, waist and ankle, waking hours of 7 days, 60 s) | Total (PAEE, compared to ankle counts): *r* = 0.38  Total (PAEE, compared to waist counts): *r* = 0.42  Moderate-to-vigorous (PAEE, compared to ankle counts): *r* = 0.42  Moderate-to-vigorous (PAEE, compared to waist counts): *r* = 0.48 | 2–  2–  2–  2– | None |
| CHAMPS  Modified English version  Giles et al. [84] | N_HEPA(frequency)_ = 31; n_HEPA(PAEE)_ = 38; n_walking(frequency)_ = 34; n_walking(PAEE)_ = 44 | Pedometer (Yamax SW700, waist, waking hours of 7 days) | *1st timepoint*  HEPA (PAEE): ρ = 0.21  HEPA (frequency): ρ = 0.52  Walking (PAEE): ρ = 0.40  Walking (frequency): ρ = 0.57  *2nd timepoint*  HEPA (PAEE): ρ = 0.38  HEPA (frequency): ρ = 0.52  Walking (PAEE): ρ = 0.53  Walking (frequency): ρ = 0.60 | 3–  3+  1–  1–  3–  3+  1–  1– | No rating was given due to the small sample size; different levels of quality were given |
| EPIC  English version  Cust et al. [93] | 182 | Accelerometer (Actigraph model 7164, hip, three times for waking hours of 7 days [4 days], 60 s, Swartz et al. [141])  Questionnaire (Friedenreich Lifetime Total Physical Activity Questionnaire) | Total (including occupational; index): ρ = 0.29 [0.15–0.42]  Total (excluding occupational; PAEE): ρ = 0.21 [0.07–0.35]  Light-to-moderate (excluding occupational): ρ = 0.19 [0.05–0.33]  Vigorous (excluding occupational): ρ = 0.23 [0.09–0.37]  Total (excluding occupational; PAEE): d̄ = 66.4, LOA = – 66.7 to 199.6 (MET-h/week)  Total (excluding occupational; PAEE): ρ = 0.26 [0.11–0.39]  Light-to-moderate (excluding occupational): ρ = 0.26 [0.12–0.39]  Vigorous (excluding occupational): ρ = 0.40 [0.27–0.52]  Recreational (PAEE): ρ = 0.21 [0.07–0.34]  Household (PAEE): ρ = 0.46 [0.34–0.57] | 1–  2–  1–  1–  3–  3–  3–  3–  3– | Results for LOA, vigorous, light-to-moderate, recreational and household-releated PA were not included |
| FPACQ  Flemish version  Matton et al. [87] | 49 | Accelerometer + 7-day activity record (RT3 Triaxial Research Tracker, hip, waking hours of 7 days, 60 s) | *Women*  Total (duration [Tactl]): *r* = 0.28  Total (PAL): *r* = 0.50  TEE: *r* = 0.85  Sports (duration)^c^: *r* = 0.38  Transport (duration)^c^: *r* = 0.52  *Men*  Total (duration [Tactl]): *r* = 0.33  Total (PAL): *r* = 0.39  TEE: *r* = 0.55  Sports (duration)^c^: *r* = 0.66  Transport (duration)^c^: *r* = 0.55 | 1–  2+  Not assigned  1–  1+  1 –  2–  Not assigned  1+  1+ | Levels of quality for TEE were assigned; results for total PA (duration), sports, and transport were not included |
| IPAQ-SF  Chinese version  Deng et al. [89] | 224 | Pedometer (Yamax SW-200, waking hours of 7 days [7 days]) | Total (PAEE): ρ = 0.33  Moderate (PAEE): ρ = 0.08  Vigorous (PAEE): ρ = - 0.12  Walking (PAEE): ρ = 0.58  Sitting: ρ = 0.005 | 3–  3–  3–  1– | Different levels of quality were given |
| Modified Baecke  Dutch version  Pols et al. [86] | n_accelerometer_ = 28, n_diary_ = 29 | Accelerometer (Caltrac, waist, waking hours of 1 day)  Diary (Four times for 3 days) | Total: *r* = 0.22  Total: *r* = 0.51 | 1–  3– | No rating was given due to the small sample size; accelerometer results were not included |
| OA-ESI  English version  O’Brien-Cousins [95] | 327 | Questionnaire (7-day recall checklist) | Total (PAEE; LTPA compared to lifelong activity): *r* = 0.45  Total (PAEE; LTPA compared to active days per week): *r* = 0.49 | 3–  3– | None |
| PAQ-EJ  Japanese version  Yasunaga et al. [96] | 147 | Accelerometer (Kenz Lifecorder, waist, 1 month, 4 s) | Total (PAEE, compared to steps/day): ρ = 0.41  Light (PAEE): ρ = 0.28  Moderate-to-vigorous (PAEE): ρ = 0.53 | 3+  1–  1+ | Different levels of quality were given |
| PASE  Dutch version  Schuit et al. [83] | 21 | DLW (2 weeks) | Total (compared to PAR): ρ = 0.68 [0.35–0.86]  Total (compared to residuals of EE [EE-RMR]): ρ = 0.58 [0.50–0.81] | 1–  1– | No rating was given due to the small sample size |
| PASE  English version  Harada et al. [91] | n_ankle_ = 56, n_waist_ = 78 | Accelerometer (Mini-Logger Series 2000, waist and ankle, waking hours of 7 days, 60 s) | Total (compared to ankle counts): *r* = 0.59  Total (compared to waist counts): *r* = 0.52 | 1+  1+ | Different levels of quality were given |
| PASE  Japanese version  Hagiwara et al. [97] | 200  325 | Accelerometer (Life Corder, waist, 3 days)  Questionnaire (JALSPAQ) | Total (compared to walking steps): ρ = 0.17  Total (compared to EE): ρ = 0.16  Total: ρ = 0.48 | 3–  1–  3– | Different levels of quality were given |
| Pre-EPIC  Dutch version  Pols et al. [86] | n_accelerometer_ = 28, n_diary_ = 29 | Accelerometer (Caltrac, waist, waking hours of 1 day)  Diary (Four times for 3 days) | TEE: *r* = 0.22  TEE: *r* = 0.64 | Not assigned  Not assigned | Levels of quality for TEE were assigned; accelerometer results were not included |
| SBAS  English version  Taylor-Piliae et al. [99] | 1010 | Interview (Stanford Seven-Day Physical Activity Recall) | Total: Significant trend across groups  Classification (active/passive): Sensitivity = 73%, Specificity = 61% | not rated | None |
| Self-administered PAQ  Swedish version  Orsini et al. [98] | 116 | Accelerometer (Actigraph MTI model 7164, waking hours of 7 days [4 days], Matthews et al. [140])  Diary (7 days [4 days]) | Total: CCC = 0.38 [0.22–0.54]  Total: slope = 0.38 [0.20–0.55]  Total: CCC = 0.64 [0.45–0.83]  Total: slope = 0.60 [0.39–0.81]  Walking/cycling and LT exercise (PAEE): CCC = 0.42 [0.22–0.62]  Walking/cycling and LT exercise (PAEE): slope = 0.38 [0.18–0.58]  LT Inactivity: CCC = 0.52 [0.36–0.69]  LT Inactivity: slope = 0.46 [0.30–0.63] | 2–  2–  3–  3–  3–  3– | Different ratings were given |
| Self-administered PAQ  Swedish version  Norman et al. [75] | 111 | Diary (Two times for 7 days [7 days]) | Total (crude score; PAEE): ρ = 0.22  Total (corrected score; PAEE): ρ = 0.53  Occupation (PAEE): ρ = 0.38  Housework (PAEE): ρ = 0.58  Walking/bicycling/exercise (PAEE): ρ = 0.37  Watching TV/reading (PAEE): ρ = 0.49 | 3–  3–  3–  3–  3– | This study was not included in the previous review but found during the reference check of the update |

*CCC* concordance correlation coefficient, *CHAMPS* Community Health Activities Model Program for Seniors, *d̄* change in the mean, *DLW* doubly labeled water, *EE* energy expenditure, *EPIC* European Prospective Investigation into Cancer, *FPACQ* Flemish Physical Activity Computerized Questionnaire, *h* hours, *HEPA* health enhancing physical activity, *IPAQ-SF* International Physical Activity Questionnaire – short-form, *JALSPAQ* Japan Arteriosclerosis Longitudinal Study Physical Activity Questionnaire, *LOA* limits of agreement, *LT*leisure time, *LTPA* leisure time physical activity, *MET* metabolic equivalent, *OA-ESI* Older Adult Exercise Status Inventory, *PA* physical activity*, PAEE* physical activity energy expenditure, *PAL* physical activity level, *PAQ* Physical Activity Questionnaire, *PAQ-EJ* Physical Activity Questionnaire for Elderly Japanese*, PAR* physical activity ratio, *PASE* Physical Activity Scale for the Elderly, *Pre-EPIC* Questionnaire preceding EPIC, *ρ* Spearman correlation coefficient, *r* Pearson correlation coefficient, *RMR* resting metabolic rate, *s* second, *SBAS* Stanford Brief Activity Survey, *TEE* total energy expenditure

^a^Two studies (i.e., Norman et al. [75]; Loland [76]) were not included in the previous review, although published prior to September 2008. The results of the two studies are shown together with previously included studies to allow comparisons

^b^As described in Sect. 2.5, the quality of the individual study was evaluated per questionnaire and construct/dimension of PA and can be either very good (1), adequate (2), doubtful (3) or inadequate (4). Additionally, the reported results were rated (i.e., sufficient [+], insufficient [–]) as described in Sect. 2.4

^c^The quality was considered as very good because the mean acceleration within each domain (e.g., measured acceleration in leisure time) was used for the comparison
